# Exploring metal bioaccumulation ability of boreal white-rot fungi on fiberbank material

**DOI:** 10.1080/21655979.2025.2507539

**Published:** 2025-05-26

**Authors:** Burcu Hacıoğlu, Gabriela Paladino, Mattias Edman, Alireza Eivazi, Erik Hedenström

**Affiliations:** a Department of Natural Sciences, Design and Sustainable Development, Mid Sweden University, Sundsvall, Sweden; b Surface and Colloid Engineering, FSCN Research Centre, Mid Sweden University, Sundsvall, Sweden

**Keywords:** Bioremediation, potentially toxic elements, heavy metals, white-rot fungi, mycoremediation, fiberbank

## Abstract

Fiberbanks are organic-rich sediment deposits in aquatic environments, primarily formed through historical pulp and paper mill activities. These deposits consist of wood-derived fibrous materials and are contaminated with potentially toxic elements (PTEs) such as vanadium, chromium, cobalt, nickel, copper, zinc, arsenic, cadmium, and lead. The leaching of these contaminants into surrounding waters poses significant environmental and health risks, impacting aquatic ecosystems and potentially entering the food chain. Effective remediation of fiberbanks is crucial, particularly in Sweden and other regions with extensive wood-pulping industries. This study aims to evaluate the bioaccumulation capacities of 26 native Swedish white-rot fungi (WRF) species for the remediation of PTEs in fiberbank material. Fiberbank samples were collected from Sundsvall’s Bay in the Baltic Sea, while the fungal species were isolated from boreal forests in Västernorrland, Sweden. The fungi were cultured on Hagem agar medium with sterilized fiberbank material as the substrate. After two months, fungal biomass was analyzed for PTE uptake using inductively coupled plasma-mass spectrometry (ICP-MS). The results revealed significant variability (*p* < 0.001) in PTE uptake among fungal species. *Phlebia tremellosa* consistently demonstrated the highest bioconcentration factors for analyzed elements, with values for V (0.39), Cr (0.10), Co (1.81), Cu (1.54), Pb (1.65), Ni (1.28), As (0.83), Zn (3.61), and Cd (5.56). Other species, including *Laetiporus sulphureus* (0.09–4.78), *Hymenochaete tabacina* (0.08–4.52), and *Diplomitoporus crustulinus* (0.08–4.48), also exhibited significant bioremediation potential. These findings highlight the potential of native WRF species for PTEs remediation in fiberbanks and provide a foundation for mycoremediation strategies in contaminated environments.

## 1. Introduction

Wood-derived fibers and chemical byproducts from the pulp and paper industry were historically discharged into nearby aquatic environments in Sweden prior to the implementation of stricter regulations in 1969. These discharges led to the formation of what is commonly referred to in Scandinavian literature as ‘fiberbank.’ Fiberbank consists of organic-rich fibrous materials, frequently contaminated with various environmental pollutants, including potentially toxic elements (PTEs). Additionally, fiber-rich sediments, along with natural mineral sediments, are often found in proximity to fiberbank in affected water bodies [1,2,3,4,5]. Fiberbank suffer from diminished levels of dissolved oxygen due to the decomposition of their organic matter, resulting in the generation of toxic methylmercury as well as greenhouse gases such as methane and carbon dioxide. The cumulative volume of fiberbank in Sweden potentially contributes to approximately 7% of the nation’s total greenhouse gas emissions [6–8]. In Sweden, including the Baltic Sea, fiberbank and fiber-rich sediments cover an estimated surface area of 2,500,000 m^2^ and 26,500,000 m^2^, respectively [9,10]. This issue extends beyond Sweden, affecting other countries such as Finland, Norway, Canada, and the USA, where water bodies are similarly contaminated with these materials [2,11].

There could be many reasons for fiberbank to be contaminated by PTEs. Pulp and paper mills have historically used processes that could involve PTEs, such as Hg and Cd, as part of their operations. Toxic elements encompassing Pb, Cd, Cr, Cu, Ni, Zn, Co, Hg, and As were discharged into the aquatic environment alongside pyrite ash from outdated sulfite-cooking processes. PTEs introduced into the water through industrial discharges can settle into these deposits [1,5–9].

PTEs in fiberbanks pose serious environmental and health risks. These elements can leach into water, impacting aquatic ecosystems and entering the human food chain through fish and other organisms [6]. Ecologically, PTEs contamination threatens biodiversity and leads to bioaccumulation in the food chain, while for humans, it presents direct and indirect health hazards through contaminated food and water. Effective remediation, continuous monitoring, and sustained long-term management are vital for mitigating environmental degradation and safeguarding public health [7,8].

Fungi have been recognized for their potential to bioremediate PTEs from the environment through a process called mycoremediation, which utilizes fungi’s natural ability to degrade, absorb, or transform hazardous substances into less harmful forms [12,13]. Fungi can accumulate toxic elements within their cellular structures, taking up and concentrating pollutants in their biomass [14]. Additionally, they bind PTEs on their cell surfaces through physicochemical processes, where cell wall components like chitin and chitosan biopolymers can eliminate metal ions effectively [15]. Fungi also convert PTEs into stable mineral forms, reducing their bioavailability, and, in some cases, alter the oxidation state of metals, making them less toxic [16].

White rot fungi (WRF) are a group of fungi known for their unique ability to decompose lignin, a complex and highly resistant component of plant cell walls. This ability distinguishes them from most other fungi and makes them pivotal in the natural process of wood decay. They play a crucial role in forest ecosystems by breaking down dead organic matter and recycling nutrients [12,17]. WRF are explored for their ability to degrade environmental pollutants, such as pharmaceutically active compounds, polycyclic aromatic hydrocarbons, herbicides, and PTEs, through processes like bioaccumulation and transformation [18,19]. WRF produce extracellular oxidative enzymes which can modify or detoxify PTEs. The enzymatic systems of WRF can alter the oxidation state of various metals, which can transform metals into less toxic forms or immobilize them, making them less bioavailable and less harmful [20,21]. Similar to other fungi, WRF can absorb and accumulate PTEs from their environment. This occurs through ion exchange, complexation, and precipitation mechanisms involving the fungal cell walls [15,18].

Managing and remediating contaminated fiberbanks is a complex challenge that requires a thorough assessment of contamination levels, evaluation of potential environmental and public health risks, and selection of appropriate cleanup methods, such as capping, removal, or natural attenuation [4]. Fiberbank and fiber-rich sediments are unique with very different characteristics from soils or other polluted sediments, given the high content of organic matter, mixed pollution, particle size, and heterogeneity. When no other attempts for bioremediation of PTEs using fungi have been published yet, exploring their mycoremediation possibilities using native Swedish WRFs is of particular interest given the lignocellulosic material present in these sediments [19].

The aim of this study is to evaluate the potential of native WRF species for the remediation of PTEs in fiberbank materials. To achieve this, the study assessed the PTE bioaccumulation capacities of 26 native WRF species isolated from various forests in Northern Sweden. A comprehensive screening study was conducted using fiberbank material collected from the Baltic Sea as the substrate.

## 2. Materials and methods

All research reported in this paper has been conducted in an ethical and responsible manner and is in full compliance with all relevant codes of experimentation and legislation. The fungal samples used in this study were collected from forests in Sweden. According to the regulations and guidelines governing such activities, no special permissions were required for the collection of these fungi.

### 2.1. Fiberbank collection and characterization

Fiberbank material was collected from Sundsvall Bay in the central Baltic Sea, Sweden, an area heavily impacted by emissions from industries such as the Ortviken pulp and paper factory and a nearby sawmill. According to the Swedish Geological Survey [9], five fiberbanks were identified in this region, all surrounded by fiber-rich sediments.

**Figure 1.**
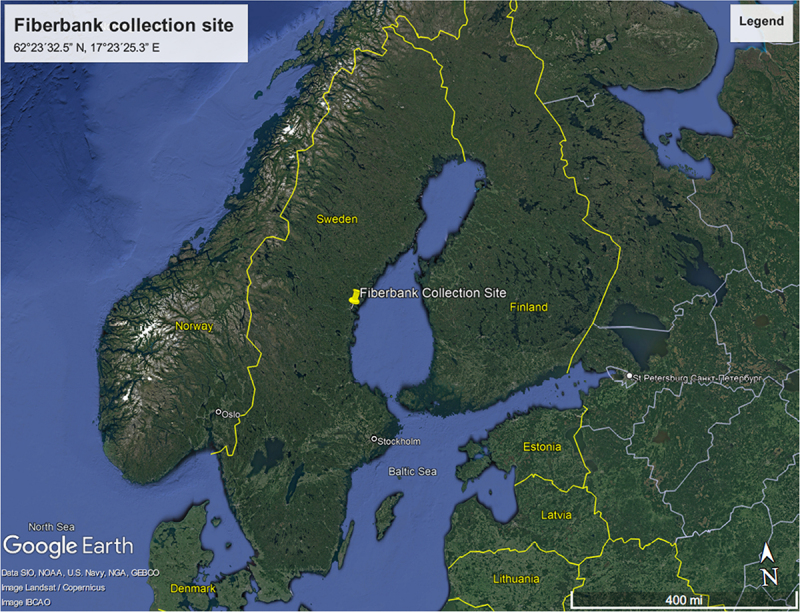
The designated site for fiberbank sampling is situated within Sundsvall Bay, along the coastline of the Bothnian Sea in Sweden. This specific location, used for mycoremediation experimental studies, is distinctly marked by a red square on the geographical map. (Source: Google Earth).

For this study, a bulk sample of 500 liters was taken from a specific fiberbank located at the eastern end of the bay, which spans 45,000 square meters and has a 6-meter-thick layer of cellulose fiber waste. The sample was collected at coordinates WGS84: 62°23´32.5” N, 17°23´25.3” E (Figure 1), from a depth of 18 meters, using a two-meter core tube sampler. The material was then dewatered over five days in a cylindrical container using gravel and geotextile.

After dewatering, the fiberbank material, including sterilized samples, was used for experimentation. For chemical analysis, samples were stored at −20 °C. Eurofins Environment Testing Sweden AB (accreditation No. 1125 ISO/IEC 17,025) performed a comprehensive environmental hazard screening, analyzing 161 parameters, including aliphatic and PAHs, VOCs, phenols, organochlorine pesticides, PCBs, herbicides, methylmercury, toxic metals, and metalloids.

Results showed a reduction in the concentrations of alkanes, aliphatic compounds, and PAHs following autoclave treatment, with only slight variations observed in potentially toxic elements (PTEs). Detailed results can be found in the supplementary materials (Table SM-1: before autoclaving, Table SM-2: after autoclaving).

### 2.2. Fungi collection and inoculation

#### 2.2.1. Fungi collection

The fungal strains utilized in this experiment were isolated from the fruiting bodies of WRF found on various natural wood substrates in the boreal forests of northern Sweden. A total of 26 species were included in the study (Table 1). These species exhibit diverse ecological functional traits, including host tree specialization and preferred stages of wood decay (primary, secondary, and late colonizers).

**Table 1.** The included 26 species of WRF and adherent GenBank accession numbers when available.

Species	GenBank
*Bjerkandera adust*a	–
*Cystostereum muraii*	–
*Diplomitoporus crustulinus*	AF343320
*Diplomitoporus lindbladii*	AJ006682
*Ganoderma applanatum*	
*Hapalopilus aurantiacus*	AY986499
*Heterobasidion annosum sensu lat.*	X70027
*Hymenochaete tabacina*	–
*Ischnoderma benzoinum*	–
*Junghuhnia collabens*	–
*Junghuhnia luteoalba*	–
*Laetiporus sulphureus*	–
*Meruliopsis taxicola*	AY787673
*Phellinus chrysoloma*	–
*Phellinus ferrugineofuscus*	JQ518285
*Phellinus nigrolimitatus*	AY558632
*Phellinus populicola*	–
*Phellinus punctatus*	–
*Phellinus viticola*	–
*Phlebia tremellosa*	–
*Phlebiopsis gigantea*	DQ320133.
*Skeletocutis odora*	–
*Stereum rugosum*	–
*Stereum sanguinolentum*	–
*Trametes hirsuta*	AB158313
*Trametes ochracea*	–

The species were first identified by a specialist based on their fruiting bodies. We employed a two-step approach to verify that the correct mycelia were isolated. First, we confirmed that the isolated mycelia exhibited clamp connections, a structural feature typical of most basidiomycete wood-decay fungi. Second, we verified that the isolated mycelium matched the fruiting body by assessing its morphological characteristics. Specifically, we evaluated the color and texture of the aerial mycelium, and in some cases, we performed microscopic examinations for additional verification following the methods of [22]. Furthermore, nine of the isolated species were independently confirmed through DNA sequencing as part of a separate project. This thorough verification process ensured the accurate identification of all fungal isolates used in the study.

#### 2.2.2. Fungi inoculation

Twenty-six fungal species were collected, cultured, and propagated on Hagem agar medium. The medium was prepared with 20 g agar (Sigma-Aldrich, United States), 5 g glucose (Sigma-Aldrich, United States, ≥99.5%), 5 g malt extract (Millipore, Germany), 0.5 g NH₄NO₃ (Supelco, United States, ≥95.0% (alkalimetric)), 0.5 g MgSO₄·7 h₂O (Sigma-Aldrich, United States, ≥99%), and 0.5 g KH₂PO₄ monobasic (Sigma-Aldrich, United States, ≥99%) per liter. The fungi were pre-cultivated for two weeks at room temperature (~21°C) before being inoculated onto the fiberbank material. The purpose of the experiment was to observe the fungi’s attraction to the fiberbank substrate and assess their ability to interact with and metabolize these materials [23–25].

**Figure 2.**
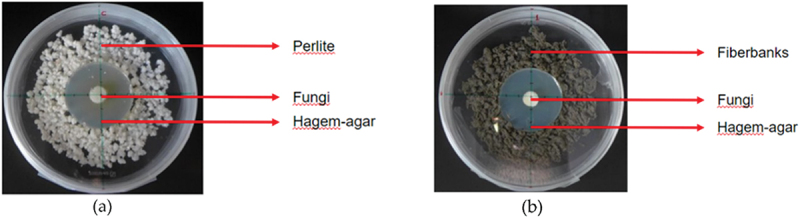
The experimental setup is illustrated through two distinct configurations as depicted in the accompanying images. Image (a) highlights the control samples, which consist of a layer of perlite surrounding a central section of Hagem agar. A disc of fungal mycelium is centrally positioned on top of the agar. In contrast, image (b) presents the treatment setup where the perlite is replaced by fiberbank material while retaining the same configuration: a central section of Hagem agar with a fungal mycelium disc placed at the center and on top of the agar. (Source: Burcu Hacıoğlu).

Inside a sterile laminar flow cabinet, a 5 cm radius disc of Hagem agar was prepared and placed at the center of a petri plate with a 16 cm diameter. Around this agar disc, sterilized fiberbank material – autoclaved at 121°C for 20 minutes – was evenly distributed to cover an area approximately 15 cm in radius. Fungal specimens from the Hagem agar plates were then aseptically transferred to the center of the agar disc using a sterile pipette with a 5 mm radius. These specimens were introduced at the center of the Hagem agar disc, positioned within the heart of the fiberbank substrate (Figure 2b). Three replicates with fiberbank substrate were prepared for each species.

For the control samples, sterilized perlite was used instead of fiberbank material. Perlite was sterilized under the same conditions as the fiberbank substrate (autoclaved at 121°C for 20 minutes) and evenly spread around the Hagem agar disc to cover an area with approximately 15 cm diameter (Figure 2a). A single replicate of perlite control was prepared for each fungal species.

### 2.3. Fungi incubation and growth in fiberbank material

After inoculation, the plates were sealed with parafilm and incubated at room temperature (~21°C) under regular daylight conditions for two months. Fungal growth progression was monitored weekly, and photographs were captured using a Leica D-LUX 6 camera. The extent of fungal expansion was quantified using ImageJ (1.53g60) software [26,27]. To maintain consistency and minimize environmental interference, photographs were taken in a controlled environment using a Puluz Photo Light Box with medium flush lighting. In ImageJ, the mycelium-covered area was carefully measured by manually outlining the fungal growth in the images, allowing for accurate calculation of expansion over time.

At the end of the two-month growth period, the mycelium was collected from the top of the Hagem agar disc using a sterile loop. The collected mycelium was then freeze-dried for 24 hours for further analysis.

### 2.4. Elemental extraction and analysis

Freeze-dried samples of the fiberbank substrate were subjected to acid digestion in sealed vessels using a microwave digestion system (Milestone-ETHOS EASY, SK-15 high-pressure rotor). Similarly, fungal mycelium collected after two months of growth underwent acid digestion in sealed vessels using the same system. One aliquot from each replicate was prepared for metal analysis. This digestion process entailed the utilization of 7.5 ml of concentrated nitric acid (HNO_3_, Supelco, United States. 65%) and 2.5 ml of hydrochloric acid (HCl, Supelco, United States, 30%). The microwave digestion involved two periods of 20 and 15 minutes each, conducted at a temperature of 210 °C.

For quality assurance in the analytical procedures, each digestion batch encompassed both a digestion blank and a certified reference material, specifically ERM-CD281 for fungi samples, and Sewage Sludge-BCR-145 R for fiberbank substrate samples. These reference materials were subjected to analogous treatment as the samples themselves. The analysis protocol adhered to the guidelines stipulated by USEPA-SW846 methods 3051A, 3052, and 6020b. An aliquot of the resulting digest was diluted with high-purity water (type 1) and supplemented with 2 µg/ml of gold (Au; 100 µg/mL in 5% HCl from VHG™, Germany) from a commercially available gold preservative stock solution for Hg (100 µg Au/mL in 5% HCl).

The quantification of elements including V, Cr, Co, Ni, Cu, Zn, As, Cd, Hg, and Pb was conducted via inductively coupled plasma-mass spectrometry (ICP-MS) utilizing an Agilent 7700 series model (Agilent Technologies Sweden AB). This analysis was performed on the prepared dilutions, employing an external calibration approach. Multielement standard solutions were appropriately diluted in a 2% v/v nitric acid/hydrochloric acid (3:1) aqueous solution, ensuring compatibility with the sample matrix and facilitating the generation of linear calibration curves exhibiting satisfactory regression values (*R* ≥ 0.98) within the required concentration ranges.

All acid reagents utilized held trace metal analytical grade (Supelco®-Merck) and were employed without additional purification steps. The multielement standard solutions were procured from Sigma-Aldrich, whereas the gold preservative solution for Hg was sourced from VHG™.

### 2.5. Statistics and data analysis

To assess the variations in PTE uptake among different fungal species, a one-way analysis of variance (ANOVA) was performed. This analysis was followed by post-hoc pairwise comparisons using the Least Significant Difference (LSD) test, with a confidence level of 95%. Before conducting these analyses, the assumptions of normality, independence of errors, and homogeneity of variances were verified. All statistical procedures were executed using RStudio (produced in 2024), specifically utilizing version 4.2.3 of the R software package.

The bioconcentration factor (BCF) was calculated to compare the bioaccumulation abilities of different WRF. The BCF is defined by the following equation:

$$\mathrm{BCF}=\frac{\mathrm{Cf}}{\mathrm{Cfb}}$$

where BCF represents the bioconcentration factor, *C_f_* denotes the concentration of the substance in the fungal tissue, and *C_fb_* is the concentration of the substance in the fiberbank material [28].

The heatmaps illustrating the BCF values for V, Cr, Co, Cu, Pb, Ni, As, Zn, and Cd were generated using the RStudio environment by hierarchical clustering, providing a more comprehensive visualization of patterns and relationships within the data.

## 3. Results and discussion

### 3.1. Growth percentage of WRF

These results regarding the percentage growth of WRF were previously published in Hacıoğlu et al. [19]. In that study, we demonstrated the potential of WRF in the bioremediation of the fiberbank´ organic pollutants, providing foundational insights into their growth dynamics and organic pollutant degradation abilities. The current study builds on that work by further exploring the bioremediation of potentially toxic elements by the same WRF species.

**Figure 3.**
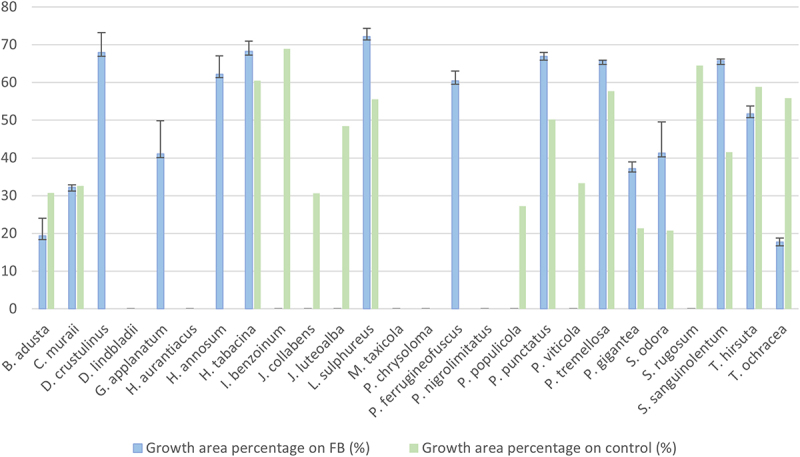
The mean (*n* = 3) growth area percentages (%) of fungal mycelia in control (perlite) and fiberbank treatments. The average percentage of mycelial growth area relative to the total plate area is calculated using the formula: growth area (%) = (Growth area of mycelium/total area of the petri dish) x100 (The petri dish has a diameter of 16 cm). Values are the mean of three replicates. Instances of zero values are indicative of where growth is confined solely to the 5 cm-Hagem-agar, with no extension over the fiberbank or perlite substrates [19].

Fifteen species of white-rot fungi (WRF), namely *B. adusta*, *C. muraii*, *D. crustulinus*, *G. applanatum*, *H. annosum*, *H. tabacina*, *L. sulphureus*, *P. ferrugineofuscus*, *P. punctatus*, *P. tremellosa*, *P. gigantea*, *S. odora*, *S. sanguinolentum*, *T. hirsuta*, and *T. ochracea*, demonstrated the ability to grow beyond the Hagem agar disc area, extending over the fiberbank material (Figure 3).

When mycelial growth did not extend beyond the Hagem-agar, it was recorded as zero, as the fungi did not encounter the fiberbank or perlite material. The growth of the following eleven species of WRF was restricted to the Hagem agar disc, and their growth over the fiberbank area was measured as zero: *D. lindbladii*, *H. aurantiacus*, *I. benzoinum*, *J. collabens*, *J. luteoalba*, *M. taxicola*, *P. chrysoloma*, *P. nigrolimitatus*, *P. populicola*, *P. viticola*, and *S. rugosum*.

The large variation in mycelial growth among fungal species may depend on several factors, including the nutritional composition of the agar media. Camenzind et al. [29] noted that some fungi may grow better in nutrient-limited media than in nutrient-rich environments. Slower-growing species may remain confined to Hagem agar, while faster-growing species extend beyond in search of more nutrients. Additionally, mycelial growth might stop when encountering fiberbank substrates due to potential toxicity, as tolerance to organic pollutants and heavy metals varies between species [19,23], which could explain the diverse growth patterns observed.

### 3.2. Bioaccumulation of PTEs

Only the WRF species that demonstrated growth in fiberbank material were assessed for their ability to accumulate PTEs. Species as *D. lindbladii*, *H. aurantiacus*, *I. benzoinum*, *J. collabens*, *J. luteoalba*, *M. taxicola*, *P. chrysoloma*, *P. nigrolimitatus*, *P. populicola*, *P. viticola*, and *S. rugosum*, which showed no growth, were excluded from further degradation studies. This selective approach ensures that the focus remains on evaluating the bioremediation potential of fungi capable of adapting to and utilizing the nutrients within the fiberbank material.

The results of the ANOVA test reveal statistically significant differences in the uptake of various elements among the fungal species. The p-values for V, Cr, Co, Ni, Cu, Zn, Cd, As, and Pb are all below 0.001, indicating a highly significant variation in element accumulation across the species studied. Full results from ANOVA including F-value, exact p-value, and the degree of freedom can be found in the supplementary material (Table SM-5).

The precise concentrations of each element in the fungal tissue for the fungi species and BCF of each targeted elements for WRF are provided in the supplementary material, specifically in Table SM-3 and Table SM-4.

**Figure 4.**
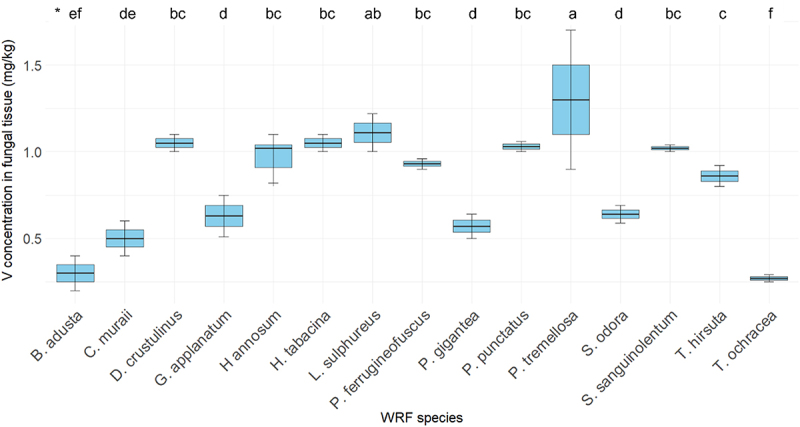
Elemental concentration of V (*n* = 3) in fungal tissue (*p* < 0.001). (*) the letters indicate the outcomes derived from the post-hoc LSD test.

The concentration of V within fungal tissues is illustrated in Figure 4. The V concentration in the fiberbank substrate was measured to be 3.35 mg/kg. Among the fungal species examined, *P. tremellosa* exhibited the highest concentration of V, measuring 1.3 mg/kg. Other species, including *L. sulphureus, D. crustulinus, H. tabacina, P. punctatus, H. annosum*, and *S. sanguinolentum*, also demonstrated significant V concentrations, each exceeding 1 mg/kg.

**Figure 5.**
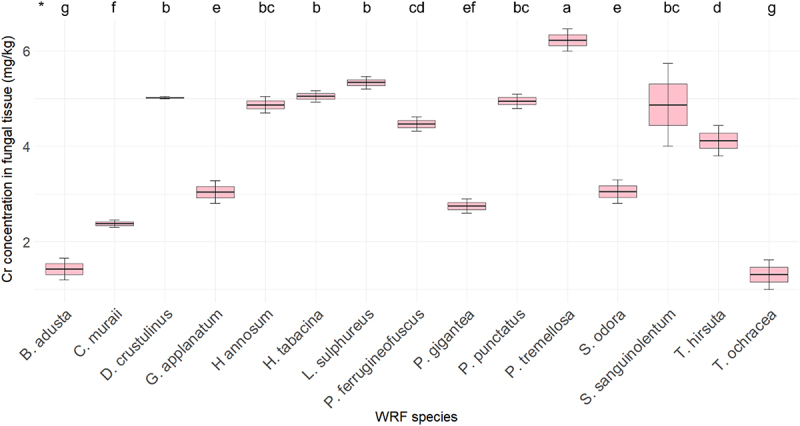
Elemental concentration of Cr (*n*=3) in fungal tissue (*p*<0.001). (*) the letters indicate the outcomes derived from the post-hoc LSD test.

The concentration of Cr within fungal tissues is illustrated in Figure 5. The concentration of Cr in the fiberbank material was measured at 62.49 mg/kg. Among the fungal species analyzed, *P. tremellosa* displayed the highest Cr uptake, with a concentration of 6.23 mg/kg. Following this, *L. sulphureus, H. tabacina*, and *D. crustulinus* exhibited Cr concentrations exceeding 5 mg/kg within their tissues. Additionally, *P. punctatus, H. annosum, S. sanguinolentum, P. ferrugineofuscus*, and *T. hirsuta* had Cr concentrations ranging from 4.95 to 4.12 mg/kg.

**Figure 6.**
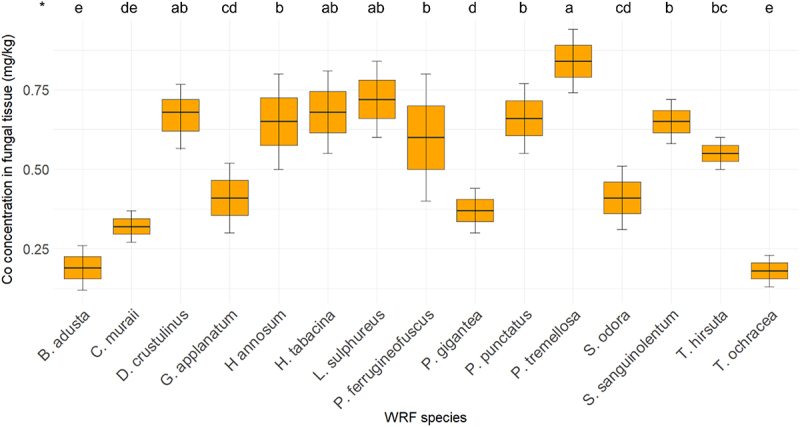
Elemental concentration of Co (*n* = 3) in fungal tissue (*p* < 0.001). (*) the letters indicate the outcomes derived from the post-hoc LSD test.

Figure 6 presents the concentration of Co in WRF tissue. The Co concentration in the fiberbank material was found to be 0.47 mg/kg. Among the fungi analyzed, *P. tremellosa* exhibited the highest Co concentration at 0.84 mg/kg. This was followed by *L. sulphureus* with a Co concentration of 0.72 mg/kg. Both *D. crustulinus* and *H. tabacina* demonstrated identical concentrations of 0.68 mg/kg. Additionally, *P. punctatus, H. annosum, S. sanguinolentum*, and *P. ferrugineofuscus* displayed Co concentrations exceeding 0.5 mg/kg.

**Figure 7.**
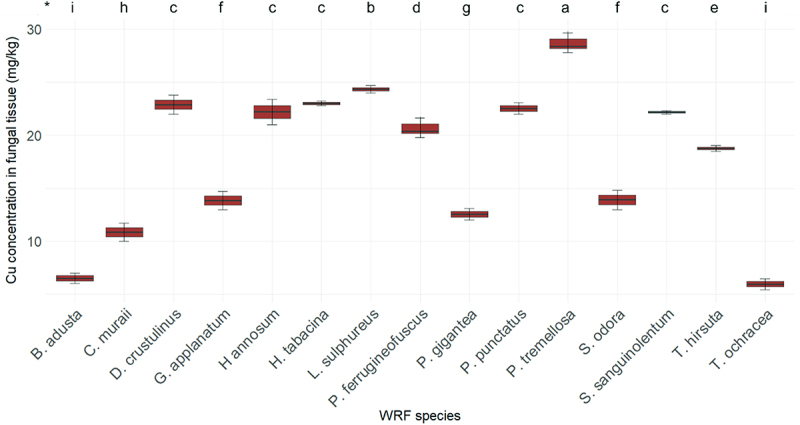
Elemental concentration of Cu (*n* = 3) in fungal tissue (*p* < 0.001). (*) the letters indicate the outcomes derived from the post-hoc LSD test.

Figure 7 illustrates the Cu concentrations in various fungal species. The Cu concentration in the fiberbank substrate is measured at 18.41 mg/kg. Among the fungi examined, *P. tremellosa* shows the highest Cu concentration at 28.39 mg/kg. This is followed by *L. sulphureus* with a concentration of 24.24 mg/kg and *H. tabacina* with 23.01 mg/kg. The species *D. crustulinus, P. punctatus, H. annosum*, and *S. sanguinolentum* exhibit similar Cu concentrations, ranging from 22.89 to 22.17 mg/kg.

**Figure 8.**
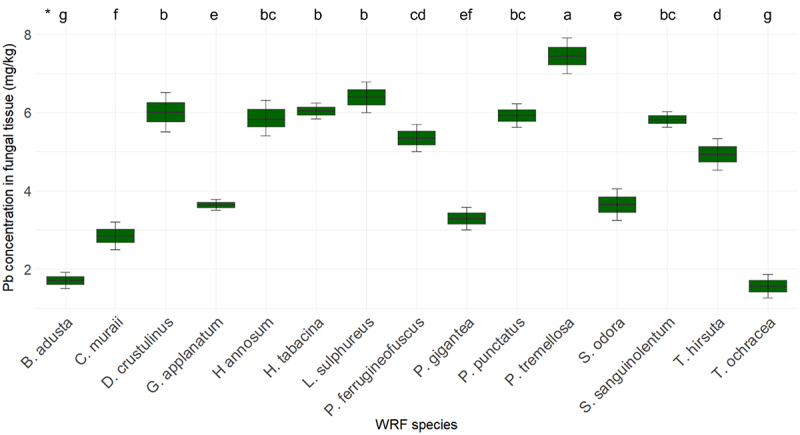
Elemental concentration of Pb (*n* = 3) in fungal tissue (*p* < 0.001). (*) the letters indicate the outcomes derived from the post-hoc LSD test.

Figure 8 illustrates the concentration of Pb in fungal tissues. The Pb concentration in the fiberbank material is measured at 4.51 mg/kg. Among the fungi studied, *P. tremellosa* exhibits the highest Pb concentration at 7.45 mg/kg. This is followed by *L. sulphureus* with a Pb concentration of 6.39 mg/kg, *H. tabacina* with 6.04 mg/kg, and *D. crustulinus* with 6.01 mg/kg. The Pb concentrations in *P. punctatus, H. annosum, S. sanguinolentum*, and *P. ferrugineofuscus* range from 5.92 mg/kg to 5.35 mg/kg.

**Figure 9.**
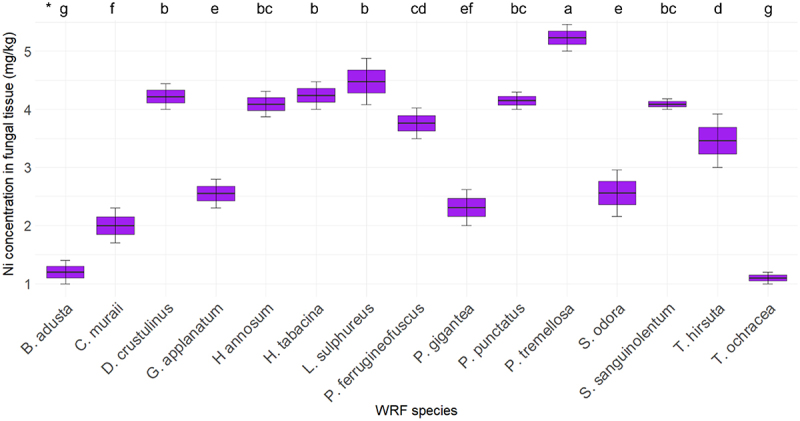
Elemental concentration of Ni (*n* = 3) in fungal tissue (*p* < 0.001). (*) the letters indicate the outcomes derived from the post-hoc LSD test.

Figure 9 depicts the concentrations of Ni in various fungal species. The Ni concentration in the fiberbank substrate is recorded at 4.09 mg/kg. Among the fungi, *P. tremellosa* shows the highest Ni concentration at 5.23 mg/kg. *L. sulphureus* follows with a concentration of 4.48 mg/kg. *H. tabacina* and *D. crustulinus* have Ni concentrations of 4.24 mg/kg and 4.22 mg/kg, respectively. *P. punctatus* has a Ni concentration of 4.15 mg/kg. Both *H. annosum* and *S. sanguinolentum* exhibit Ni concentrations equal to the substrate level, at 4.09 mg/kg.

**Figure 10.**
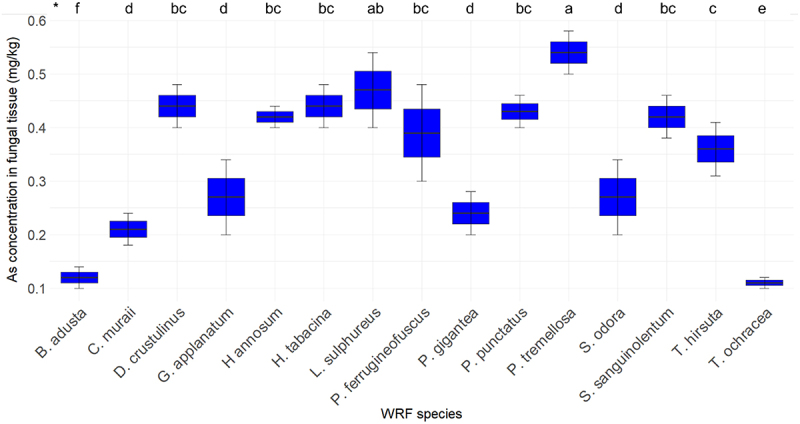
Elemental concentration of as (*n* = 3) in fungal tissue (*p* < 0.001). (*) the letters indicate the outcomes derived from the post-hoc LSD test.

Figure 10 depicts the As concentration in various fungi. The As concentration in the fiberbank material was measured at 0.65 mg/kg. Among the fungi, *P. tremellosa* exhibited the highest As concentration at 0.54 mg/kg, followed by *L. sulphureus* with 0.47 mg/kg. Both *D. crustulinus* and *H. tabacina* displayed identical As concentrations of 0.44 mg/kg. *P. punctatus*, *H. annosum*, and *S. sanguinolentum* demonstrated As concentrations ranging from 0.43 to 0.42 mg/kg.

**Figure 11.**
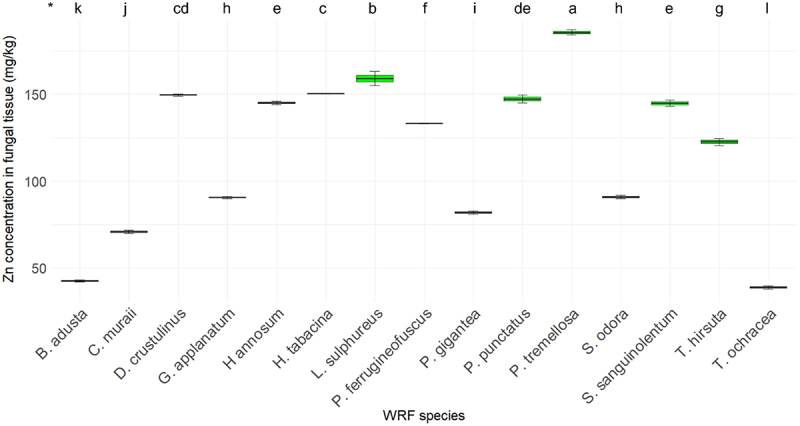
Elemental concentration of Zn (*n* = 3) in fungal tissue (*p* < 0.001). (*) the letters indicate the outcomes derived from the post-hoc LSD test.

Figure 11 illustrates the Zn concentration in various fungi. The Zn concentration in the fiberbank material is 51.44 mg/kg. Among the nine elements analyzed, Zn exhibited the highest range of concentrations, spanning from 185.52 mg/kg to 28 mg/kg. *P. tremellosa* displayed the highest Zn concentration at 185.52 mg/kg. Following this, *L. sulphureus* had a concentration of 159 mg/kg, and *H. tabacina* had 150.32 mg/kg. The Zn concentration in *D. crustulinus* was 149.58 mg/kg, in *P. punctatus* it was 147.3 mg/kg, in *H. annosum* it was 145.01 mg/kg, and in *S. sanguinolentum* it was 144.86 mg/kg.

**Figure 12.**
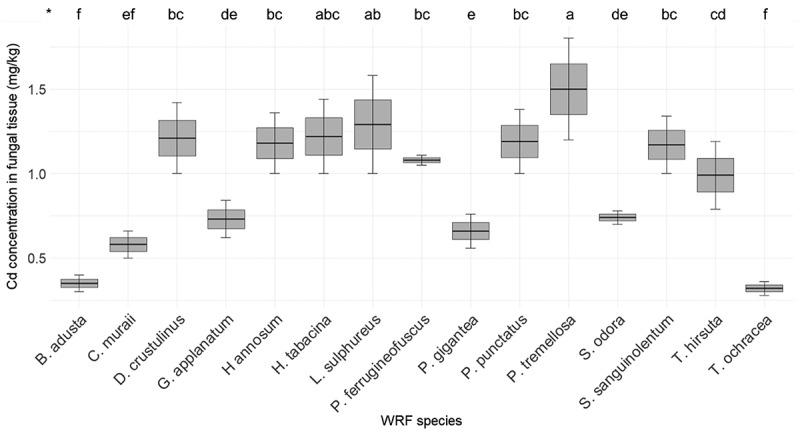
Elemental concentration of Cd (*n* = 3) in fungal tissue (*p* < 0.001). (*) the letters indicate the outcomes derived from the post-hoc LSD test.

Figure 12 presents the Cd concentration in various fungi. The Cd concentration in the fiberbank material is 0.27 mg/kg. Among the fungi, *P. tremellosa* exhibited the highest Cd concentration at 1.5 mg/kg. This is followed by *L. sulphureus* with 1.29 mg/kg, and *H. tabacina* with 1.22 mg/kg. The Cd concentrations in *D. crustulinus*, *P. punctatus*, *H. annosum*, and *S. sanguinolentum* were 1.21 mg/kg, 1.19 mg/kg, 1.18 mg/kg, and 1.17 mg/kg, respectively. *P. ferrugineofuscus* displayed a Cd concentration of 1.08 mg/kg.

Hg concentrations were measured in the fungal tissues; however, all results were zero. Consequently, these measurements were not included in the graphical representations or the main text of the study.

**Figure 13.**
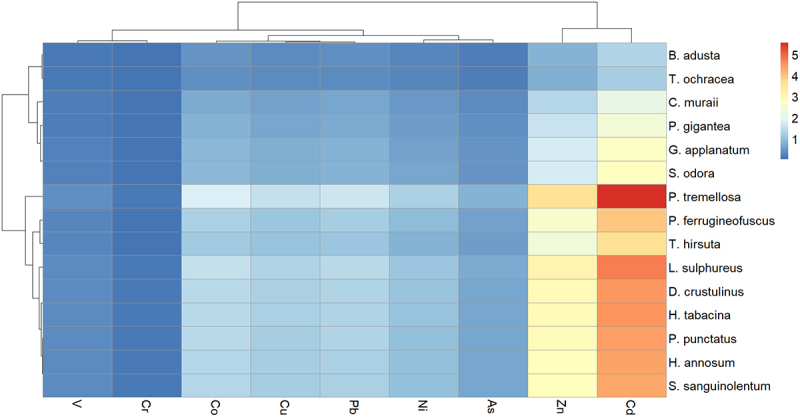
Heat map of BCF of V, Cr, Co, Cu, Pb, Ni, As, Zn, and Cd. BCF was calculated as the concentration of the elements in fungal tissue divided by the concentration of the elements in fiberbank material.

The heat map of the BCF for all nine elements is shown in Figure 13. Checking BCF is crucial since the bioaccumulation of PTEs depends on the material on which fungi grow. This allows to observe the bioaccumulation efficiency of different WRF species.

It is evident that Cd and Zn exhibit the greatest variation in accumulation among the PTEs. WRF require trace amounts of essential heavy metals like Cd and Zn for their metabolic processes [30]. In this study, Cd concentrations in WRF mycelia ranged from 0 to 1.5 mg/kg, compared to 0.27 mg/kg in the fiberbank material. Although some studies have reported higher Cd levels in fungi, such as 2.67 mg/kg in *Agaricus campestris* [31], and *L. sulphureus* had a Cd concentration of 140.86 µg/g [32] while it has 1.29 mg/kg in our study, it is crucial to compare these values with the Cd levels in the fiberbank substrate. In our findings, *P. tremellosa* (5.56), *L. sulphureus* (4.78), *D. crustulinus* (4.48), *H. tabacina* (4.52), *P. punctatus* (4.41), *H. annosum* (4.37), and *S. sanguinolentum* (4.33) demonstrated the highest BCF for Cd, highlighting their bioaccumulation capabilities.

Zn plays an important role in the lignin-modifying enzymes and metal tolerance of WRF [33]. However, beyond a certain concentration, Zn becomes toxic, and the toxicity threshold varies among different species [30]. In our study, *P. tremellosa* and *L. sulphureus* exhibited the highest BCFs of 3.61 and 3.09. Sharma et al. [34] reported a high Zn concentration in biomass at 77 mg/g by a newly isolated fungus, whereas *P. tremellosa* in our study showed a concentration in biomass of 185.52 mg/kg. However, it is important to consider the Zn concentration in the fiberbank material, which is 51.44 mg/kg, for accurate comparison. This context highlights the significant bioaccumulation capabilities of the studied WRF species in relation to the Zn content of their growth substrate.

*Phlebia tremellosa* displayed the highest BCF at 0.83 for As, suggesting capability to accumulate As from its surroundings. This is followed by *L. sulphureus* (0.72) and *D. crustulinus* (0.68). The variation in BCF among different species highlights differences in their metabolic pathways and As uptake mechanisms. In comparison with literature, similar trends are observed [35]. For instance, the bioaccumulation of As in fungal biomass (of 54 fungi species) ranged from 0.023 to 0.259 g/kg in Singh et al. [36] study while in our case it ranges from 0.1 to 0.54 mg/kg. *L. sulphureus* has been note for As bioaccumulation in other studies as in Bulam et al. [37] *L. sulphureus* collected from nature has a 0.01 mg/kg As in fungal tissue.

Ni BCF data mirrors the concentration in biomass results, with *P. tremellosa* having the highest BCF at 1.28. This suggests that it not only accumulates high levels of Ni but also concentrates it more effectively relative to its environment. Other notable species include *L. sulphureus* (1.1), *H. tabacina* (1.04), and *D. crustulinus* (1.03). These fungi show BCF values above 1, indicating efficient bioconcentration abilities. Lower BCF values are observed for species like *B. adusta* (0.29), *T. ochracea* (0.27), and *H. aurantiacus* (0.24), indicating lesser efficiency in Ni accumulation. *P. tremellosa* has been documented in other studies for its metal accumulation capabilities, which is consistent with its high Ni concentration in biomass and BCF in this study. Higher Ni concentrations have been reported in the literature, such as 69.4 mg/kg of nickel in Helvella spadicea [38], compared to 5.23 mg/kg in P. tremellosa. However, it is important to compare these results while considering the initial Ni concentration in the fiberbank material. Similarly, *L. sulphureus* is highlighted in literature for its PTEs bioremediation potential. Species such as *G. applanatum* and *P. gigantea*, which show moderate Ni uptake in this study, are also recognized in other research for their PTEs accumulation, though typically at lower efficiencies. This variance is often due to differences in fungal metabolism, environmental conditions, and substrate specificity [39]

*Phlebia tremellosa* exhibited the highest BCF for Pb at 1.65, indicating strong Pb accumulation relative to its environment. Other species with high BCF values included *L. sulphureus* (1.42), *H. tabacina* (1.34), and *D. crustulinus* (1.33), suggesting their efficient bioconcentration abilities. In some cases, the bioaccumulation factor of Pb in L. sulphureus has been reported to reach up to 42.64 [32], whereas in our study, it is limited to 1.42. Lower BCF values were observed for species such as *B. adusta* (0.38), *T. ochracea* (0.35), and *H. aurantiacus* (0.29), indicating lesser efficiency in Pb accumulation. Moderate Pb uptake was observed in species like *G. applanatum*, which has been noted in other studies for its metal accumulation capabilities, though typically with lower efficiencies than the species identified with higher BCFs [30]. These variations in Pb uptake reflect the diverse bioaccumulation capacities of different fungal species, with some being more suited for bioremediation of Pb-contaminated environments than others.

The BCF values align with the Cu concentration in biomass data, with *P. tremellosa* exhibiting the highest BCF at 1.54. This indicates that *P. tremellosa* not only accumulates significant amounts of Cu but also concentrates it efficiently relative to its environment. Other species with high BCF values include *L. sulphureus* (1.32), *H. tabacina* (1.25), and *D. crustulinus* (1.24), highlighting their strong bioconcentration abilities. Comparatively, *Pleurotus ostreatus* is known to accumulate substantial Cu, similar to *P. tremellosa* and *L. sulphureus* [30]. Additionally, *Trametes versicolor* can accumulate Cu in concentrations (in biomass) ranging from 20 to 30 mg/kg in its mycelia, supporting the high Cu uptake observed in *P. tremellosa* and *H. tabacina* [40]. These findings reinforce the potential of these fungi for Cu bioremediation.

*Phlebia tremellosa* exhibited the highest BCF for Co at 1.81, indicating its strong ability to accumulate and concentrate in biomass relative to its environment. Other species with high BCF values include *L. sulphureus* (1.55), *D. crustulinus* (1.46), and *H. tabacina* (1.46), all showing effective Co bioconcentration. The Co concentrations in biomass observed in species like *P. tremellosa, L. sulphureus, D. crustulinus, H. tabacina, P. punctatus, H. annosum*, and *S. sanguinolentum* are comparable to findings from the literature. For instance, *Ganoderma lucidum* and *Pleurotus sajor-caju* have been reported to accumulate Co in concentrations ranging from 65 to 85 mg/kg [40,41], supporting the strong Co bioaccumulation potential of these fungi.

*Phlebia tremellosa* exhibited the highest BCF for Cr at 0.1, indicating a moderate capacity for Cr uptake from the environment. Other species with relatively high BCF values include *L. sulphureus* (0.09), *D crustulinus* (0.08), and *H. tabacina* (0.08). The Cr concentration in biomass and BCF by these WRF species is lower than what has been reported in the literature, as *Agaricus bitorquis* 250 mg/g for Cr(III) and 227.27 mg/g for Cr(VI) in another study [42] which may be attributed to the formation of Cr complexes with organic and inorganic ligands that reduce their bioavailability. Organic matter in soils can bind to Cr, limiting its mobility and availability to organisms. In environments like fiberbank, which have high organic matter content, more Cr may be adsorbed, further decreasing its bioavailability to fungi [43,44]. This highlights the importance of considering environmental factors when assessing the bioremediation potential of fungi for Cr.

*Phlebia tremellosa* exhibited the highest BCF for V at 0.39, suggesting it effectively accumulates V relative to its environment. Regarding the concentration of V in fungal tissue, some studies report significantly higher levels than those observed in the WRF in this study. For instance, *B. adusta* grown on a medium amended with 10 mm VOSO₄ accumulated up to 37.4 mg/g of V [45], whereas in our study, V concentration in *B. adusta* was only 0.3 mg/kg. This highlights the importance of considering the initial concentration, as well as the differences between a model media and real organic material. The bioavailability of V in fiberbank material may generally be low, due to the presence of less bioavailable V forms and the influence of soil properties that limit its uptake [46,47]. Other species with relatively high BCF values include *L. sulphureus* (0.33), *D. crustulinus* (0.31), and *H. tabacina* (0.31). The differences in BCF values among the species highlight the varying capacities of white-rot fungi to uptake V from fiberbank environments, likely influenced by both the chemical forms of V present and the environmental conditions.

## 4. Conclusion

This study aimed to analyze the bioaccumulation capabilities of various WRF for multiple PTEs from fiberbank material. The results indicate variability in the uptake and BCF across different fungal species and elements.

In this study, *P. tremellosa* demonstrated exceptional potential for bioremediation of PTEs from fiberbank materials, consistently showing the highest bioaccumulation factors for metals such as V, Cr, Co, Cu, Pb, Ni, Zn, and Cd. Its ability to accumulate these elements in significant concentrations highlights its suitability for remediating fiberbank, where diverse metal contaminants are prevalent.

The strong bioaccumulation capacity of *P. tremellosa*, particularly for elements like Cu and Zn, suggests it could be effectively applied to clean contaminated sites. However, scaling up bioremediation with fungi will require optimizing cultivation methods, improving inoculation techniques, and addressing environmental factors that affect remediation efficiency in real-world conditions.

Future research should focus on large-scale deployment strategies, including enhancing metal uptake through bioengineering and addressing the disposal or recovery of metal-laden fungal biomass. With further development, *P. tremellosa* holds promise for contributing to sustainable solutions for PTE contamination in fiberbank and beyond.

## Supplementary Material

Supplementary material.docx

## Data Availability

The authors confirm that the data supporting the findings of this study are available within the article [and/or] its supplementary materials.

## References

[1] Dahlberg AK, Apler A, Vogel L, et al. Persistent organic pollutants in wood fiber–contaminated sediments from the Baltic Sea. J Soils Sediments. 2020;20(5):2471–17. doi: 10.1007/s11368-020-02610-6

[2] Göransson G, Apler A, Dahlberg AK, et al. Assessing the risk of contaminant dispersion from fibrous sediments of industrial origin. Front Mar Sci. 2021;8:729243. doi: 10.3389/fmars.2021.729243

[3] Lehoux AP, Isidorova A, Collin F, et al. Extreme gas production in anthropogenic fibrous sediments: an overlooked biogenic source of greenhouse gas emissions. Sci Total Environ. 2021;781:146772.

[4] Haller H, Paladino G, Dupaul G, et al. Polluted lignocellulose-bearing sediments as a resource for marketable goods—a review of potential technologies for biochemical and thermochemical processing and remediation. Clean Technol Environ Policy. 2023;25(2):409–425.

[5] Löfroth H, O’Regan M, Snowball I, et al. Challenges in slope stability assessment of contaminated fibrous sediments along the northern Baltic coast of Sweden. Eng Geol. 2021;289:106190. doi: 10.1016/j.enggeo.2021.106190

[6] Apler A, Snowball I, Josefsson S. Dispersal of cellulose fibers and metals from contaminated sediments of industrial origin in an estuary. Environ Pollut. 2020;266:115182. doi: 10.1016/j.envpol.2020.115182 32673976

[7] Frogner-Kockum P, Kononets M, Apler A, et al. Less metal fluxes than expected from fibrous marine sediments. Mar Pollut Bull. 2020;150:110750. doi: 10.1016/j.marpolbul.2019.110750 31780085

[8] Regnell O, Elert M, Höglund LO, et al. Linking cellulose fiber sediment methyl mercury levels to organic matter decay and major element composition. Ambio. 2014 Nov;43(7):878–890. doi: 10.1007/s13280-013-0487-2 24420263 PMC4190148

[9] Apler A, Nyberg J, Jönsson K, et al. Kartläggning avfiberhaltiga sediment längs Västernorrlands kust.Sweden: Sveriges geologiska undersökning. Länsstyrelsen i Västernorrland. 2014. (SGU-rapport 2014:16).

[10] Norrlin J, Josefsson S, Larsson O, et al. Kartläggning och riskklassning av fiberbankar I Norrland. SGU-rapport. 2016;21:177.

[11] Hoffman E, Alimohammadi M, Lyons J, et al. Characterization and spatial distribution of organic-contaminated sediment derived from historical industrial effluents. Environ Monit Assess. 2019;191(9):1–19. doi: 10.1007/s10661-019-7763-y 31444645

[12] Harms H, Schlosser D, Wick LY. Untapped potential: exploiting fungi in bioremediation of hazardous chemicals. Nat Rev Microbiol. 2011;9(3):177–192. doi: 10.1038/nrmicro2519 21297669

[13] Singh RK, Tripathi R, Ranjan A, et al. Fungi as potential candidates for bioremediation. In: Singh P., Kumar R. Borthakur, editors. Abatement of environmental pollutants. Netherlands: Elsevier; 2020. p. 177–191.

[14] Falandysz J, Treu R. Fungi and environmental pollution. J Environ Sci Health, Part B. 2017;52(3):147–147. doi: 10.1080/03601234.2017.1261535 28121275

[15] Zare P, Giyahchi M, Moghimi H. Mycosorption and mycoremediation: fungi as the tools for heavy metal removal. In: Uppuluri, K.B. Selvasembian, R., editors. Bioprospecting of multi-tasking fungi for a sustainable environment. Singapore: Springer; 2024. p. 249–272.

[16] Priyadarshini E, Priyadarshini SS, Cousins BG, et al. Metal-fungus interaction: review on cellular processes underlying heavy metal detoxification and synthesis of metal nanoparticles. Chemosphere. 2021;274:129976. doi: 10.1016/j.chemosphere.2021.129976 33979913

[17] Echezonachi SO. Chapter 16 - the role of white rot fungi in bioremediation. In: Malik JA, editor. Microbes and microbial biotechnology for green remediation elsevier eBooks. Netherlands: Elsevier; 2022. p. 305–320.

[18] Chen L, Zhang X, Zhang M, et al. Removal of heavy-metal pollutants by white rot fungi: mechanisms, achievements, and perspectives. J Cleaner Prod. 2022;354:131681. doi: 10.1016/j.jclepro.2022.131681

[19] Hacıoğlu B, Dupaul G, Paladino G, et al. Unlocking the biodegradative potential of native white-rot fungi: a comparative study of fiberbank organic pollutant mycoremediation. Bioengineered. 2024;15(1):2396642. doi: 10.1080/21655979.2024.2396642 39219315 PMC11370975

[20] Silva RRD. Potential of white-rot fungi for bioremediation. Rev Bras Gest Amb Sustent. 2017;4(7):229–232. doi: 10.21438/rbgas.040722

[21] Sutjaritvorakul T. Biotransformation of toxic metal compounds by fungi. วารสาร วิชาการ ปทุมวัน Pathumwan Academic J. 2014;4(11):37–43.

[22] Stalpers JA. Identification of wood-inhabiting aphyllophorales in pure culture J.A. Stalpers. Baarn: Centraalbureau voor Schimmelcultures; 1978. p. 1–248.

[23] Lee H, Jang Y, Choi YS, et al. Biotechnological procedures to select white rot fungi for the degradation of PAHs. J Microbiol Methods. 2014;97:56–62. doi: 10.1016/j.mimet.2013.12.007 24374215

[24] Mohamadhasani F, Rahimi M. Growth response and mycoremediation of heavy metals by fungus Pleurotus sp. Sci Rep. 2022;12(1):19947. doi: 10.1038/s41598-022-24349-5 36402909 PMC9675861

[25] Valentín L, Oesch-Kuisma H, Steffen KT, et al. Mycoremediation of wood and soil from an old sawmill area contaminated for decades. J Hazard Mater. 2013;260:668–675. doi: 10.1016/j.jhazmat.2013.06.014 23832059

[26] Schindelin J, Arganda-Carreras I, Frise E, et al. Fiji: an open-source platform for biological-image analysis. Nat Methods. 2012;9(7):676–682. 22743772 10.1038/nmeth.2019 PMC3855844

[27] Cardini A, Pellegrino E, Del Dottore E, et al. HyLength: a semi-automated digital image analysis tool for measuring the length of roots and fungal hyphae of dense mycelia. Mycorrhiza. 2020;30(2):229–242. 32300867 10.1007/s00572-020-00956-w

[28] Melgar MJ, Alonso J, García MA. Mercury in edible mushrooms and underlying soil: bioconcentration factors and toxicological risk. Sci Total Environ. 2009;407(20):5328–5334. doi: 10.1016/j.scitotenv.2009.07.001 19631362

[29] Camenzind T, Lehmann A, Ahland J, et al. Trait‐based approaches reveal fungal adaptations to nutrient‐limiting conditions. Environ Microbiol. 2020;22(8):3548–3560. doi: 10.1111/1462-2920.15132 32558213

[30] Baldrian P. Interactions of heavy metals with white-rot fungi. Enzyme Microb Technol. 2003;32(1):78–91. doi: 10.1016/S0141-0229(02)00245-4

[31] Širić I, Humar M, Kasap A, et al. Heavy metal bioaccumulation by wild edible saprophytic and ectomycorrhizal mushrooms. Environ Sci Pollut Res. 2016;23(18):18239–18252. doi: 10.1007/s11356-016-7027-0 27272918

[32] Kryczyk A, Piotrowska J, Sito M, et al. Remediation capacity of Cd and Pb ions by mycelia of Imleria badia, Laetiporus sulphureus, and Agaricus bisporus in vitro cultures. J Environ Sci Health, Part B. 2017;52(9):617–622. doi: 10.1080/03601234.2017.1330068 28586282

[33] Sośnicka A. White-rot fungi and their lignin modifying enzymes: an effective tool to fight recalcitrant organic pollutants. Edukacja Biologiczna i Środowiskowa. 2018;3:3–9.

[34] Sharma S, Dastidar MG, Sreekrishnan TR. Zinc uptake by fungal biomass isolated from industrial wastewater. Pract Period Hazard Toxic Radioact Waste Manage. 2002;6(4):256–261. doi: 10.1061/(ASCE)1090-025X(2002)6:4(256)

[35] Srivastava PK, Vaish A, Dwivedi S, et al. Biological removal of arsenic pollution by soil fungi. Sci Total Environ. 2011;409(12):2430–2442. doi: 10.1016/j.scitotenv.2011.03.002 21459413

[36] Singh M, Srivastava PK, Verma PC, et al. Soil fungi for mycoremediation of arsenic pollution in agriculture soils. J Appl Microbiol. 2015;119(5):1278–1290. doi: 10.1111/jam.12948 26348882

[37] Bulam S, Karadeniz M, Bakir TK, et al. Assessment of total phenolic, total flavonoid, metal contents and antioxidant activities of Trametes versicolor and Laetiporus sulphureus. Acta Scientiarum Polonorum Hortorum Cultus. 2022;21(5):39–47. doi: 10.24326/asphc.2022.5.4

[38] Gadd GM. Geomycology: biogeochemical transformations of rocks, minerals, metals and radionuclides by fungi, bioweathering and bioremediation. Mycol Res. 2007;111(1):3–49. doi: 10.1016/j.mycres.2006.12.001 17307120

[39] Das N. Recovery of precious metals through biosorption — a review. Hydrometallurgy. 2010;103(1–4):180–189. doi: 10.1016/j.hydromet.2010.03.016

[40] Ramírez-Valdespino CA, Orrantia-Borunda E. Trichoderma and nanotechnology in sustainable agriculture: a review. Front Fungal Biol. 2021;2:764675. doi: 10.3389/ffunb.2021.764675 37744133 PMC10512408

[41] Hanif MA, Bhatti HN, Bhatti IA, et al. Bioaccumulation of Cr (III) and Cr (VI) by newly isolated white rot fungi: batch and column studies. Asian J Chem. 2011;23(8):3375–3383.

[42] Hanif MA, Bhatti HN. Remediation of heavy metals using easily cultivable, fast growing, and highly accumulating white rot fungi from hazardous aqueous streams. Desalin Water Treat. 2015;53(1):238–248. doi: 10.1080/19443994.2013.848413

[43] Olaniran AO, Balgobind A, Pillay B, et al. Bioavailability of heavy metals in soil: impact on microbial biodegradation of organic compounds and possible improvement strategies. Int J Mol Sci. 2013;14(5):10197–10228. doi: 10.3390/ijms140510197 23676353 PMC3676836

[44] Zulfiqar U, Haider FU, Ahmad M, et al. Chromium toxicity, speciation, and remediation strategies in soil-plant interface: a critical review. Front Plant Sci. 2023;13:1081624. doi: 10.3389/fpls.2022.1081624 36714741 PMC9880494

[45] Xu YH, Brandl H, Osterwalder S, et al. Vanadium-basidiomycete fungi interaction and its impact on vanadium biogeochemistry. Environ Int. 2019;130:104891. doi: 10.1016/j.envint.2019.06.001 31234005

[46] Larsson MA, Baken S, Smolders E, et al. Vanadium bioavailability in soils amended with blast furnace slag. J Hazard Mater. 2015;296:158–165. doi: 10.1016/j.jhazmat.2015.04.034 25917693

[47] Yang J, Wang Y, Gao X, et al. Vanadium: a review of different extraction methods to evaluate bioavailability and speciation. Minerals. 2022;12(5):642. doi: 10.3390/min12050642

