# Supplementary material for: Exploring metal bioaccumulation ability of boreal white-rot fungi on fiberbank material
Source: Bioengineered. 2025 May 26;16(1):2507539. doi: 10.1080/21655979.2025.2507539 (PMC12118429; doi:10.1080/21655979.2025.2507539)
Supplement: Supplementary material.docx [file KBIE_A_2507539_SM4889.docx]

**Exploring metal biosorption ability of Swedish boreal native white-rot fungi on fiberbank material**

Burcu Hacıoğlu ^1*^, Gabriela Paladino ^1^, Mattias Edman ^1^, Alireza Eivazi ^2^ and Erik Hedenström ^1*^

^1^ Mid Sweden University, Department of Natural Sciences, Design and Sustainable Development, Holmgatan 10, 851 70-Sundsvall, Sweden

^2^ Mid Sweden University, Surface and Colloid Engineering, FSCN Research Centre, Holmgatan 10, SE-851 70 Sundsvall, Sweden

**Table SM-1.** Dewatered fresh (before autoclave) fiberbanks analysis report from accredited external laboratory.

| **Analyte** | **Result** | **Unit** | **Measurement uncertainty** | **Metod/ref** |  |
| --- | --- | --- | --- | --- | --- |
| Torrsubstans | 0.96 | % | 5% | SS-EN 12880:2000 | b) |
| Torrsubstans | 0.97 | % | 5% | SS-EN 12880:2000 | a) |
| Alifater >C8-C10 | < 17 | mg/kg Ts | 35% | SPI 2011 | b)* |
| Alifater >C10-C12 | < 17 | mg/kg Ts | 30% | SPI 2011 | b)* |
| Alifater >C12-C16 | < 17 | mg/kg Ts | 30% | SPI 2011 | b)* |
| Alifater >C16-C35 | 2100 | mg/kg Ts | 30% | SPI 2011 | b)* |
| Aromater >C8-C10 | < 34 | mg/kg Ts | 30% | SPI 2011 | b)* |
| Aromater >C10-C16 | < 3.4 | mg/kg Ts | 20% | SPI 2011 | b)* |
| Aromater >C16-C35 | 1.7 | mg/kg Ts | 25% | SPI 2011 | b)* |
| Metylpyren/fluorantener | < 1.7 | mg/kg Ts | 25% | SPI 2011 | b)* |
| Metylkrysener/benzo(a)antracener | < 1.7 | mg/kg Ts | 25% | SPI 2011 | b)* |
| Bens(a)antracen | < 0.12 | mg/kg Ts | 25% | ISO 18287:2008 mod | b) |
| Krysen | < 0.12 | mg/kg Ts | 25% | ISO 18287:2008 mod | b) |
| Benso(b,k)fluoranten | 0.18 | mg/kg Ts | 25% | ISO 18287:2008 mod | b) |
| Benso(a)pyren | < 0.12 | mg/kg Ts | 25% | ISO 18287:2008 mod | b) |
| Indeno(1,2,3-cd)pyren | < 0.12 | mg/kg Ts | 25% | ISO 18287:2008 mod | b) |
| Dibens(a,h)antracen | < 0.12 | mg/kg Ts | 30% | ISO 18287:2008 mod | b) |
| Naftalen | 2.8 | mg/kg Ts | 25% | ISO 18287:2008 mod | b) |
| Acenaftylen | 0.18 | mg/kg Ts | 40% | ISO 18287:2008 mod | b) |
| Acenaften | 0.37 | mg/kg Ts | 25% | ISO 18287:2008 mod | b) |
| Fluoren | 0.13 | mg/kg Ts | 30% | ISO 18287:2008 mod | b) |
| Fenantren | 0.47 | mg/kg Ts | 25% | ISO 18287:2008 mod | b) |
| Antracen | < 0.12 | mg/kg Ts | 25% | ISO 18287:2008 mod | b) |
| Fluoranten | 0.31 | mg/kg Ts | 25% | ISO 18287:2008 mod | b) |
| Pyren | 0.44 | mg/kg Ts | 25% | ISO 18287:2008 mod | b) |
| Benso(g,h,i)perylen | < 0.12 | mg/kg Ts | 25% | ISO 18287:2008 mod | b) |
| Summa PAH med låg molekylvikt | 3.4 | mg/kg Ts |  |  | b) |
| Summa PAH med medelhög molekylvikt | 1.4 | mg/kg Ts |  |  | b) |
| Summa PAH med hög molekylvikt | 0.54 | mg/kg Ts |  |  | b) |
| Summa cancerogena PAH | 0.48 | mg/kg Ts |  |  | b) |
| Summa övriga PAH | 4.8 | mg/kg Ts |  |  | b) |
| Summa totala PAH16 | 5.3 | mg/kg Ts |  |  | b) |
| 1,1,1,2-Tetrakloretan | < 0.0050 | mg/kg Ts | 20% | EPA 5021 | b) |
| 1,1,1-Trikloretan | < 0.0050 | mg/kg Ts | 25% | EPA 5021 | b) |
| 1,1,2-Trikloretan | < 0.0050 | mg/kg Ts | 30% | EPA 5021 | b) |
| 1,1,2-Trikloreten | < 0.0050 | mg/kg Ts | 20% | EPA 5021 | b) |
| 1,1-Dikloretan | < 0.0050 | mg/kg Ts | 30% | EPA 5021 | b) |
| 1,1-Dikloreten | < 0.0050 | mg/kg Ts | 30% | EPA 5021 | b) |
| 1,1-Diklorpropen | < 0.0050 | mg/kg Ts | 25% | EPA 5021 | b) |
| 1,2,3-Triklorbensen | < 0.0050 | mg/kg Ts | 30% | EPA 5021 | b) |
| 1,2,3-Triklorpropan | < 0.0050 | mg/kg Ts | 25% | EPA 5021 | b) |
| 1,2,4-Triklorbensen | < 0.0050 | mg/kg Ts | 20% | EPA 5021 | b) |
| 1,2,4-Trimetylbensen | < 0.0050 | mg/kg Ts | 30% | EPA 5021 | b) |
| 1,2-Dibrometan | < 0.0050 | mg/kg Ts | 25% | EPA 5021 | b) |
| 1,2-Diklorbensen | < 0.0050 | mg/kg Ts | 15% | EPA 5021 | b) |
| 1,2-Dikloretan | < 0.0050 | mg/kg Ts | 25% | EPA 5021 | b) |
| 1,2-Diklorpropan | < 0.0050 | mg/kg Ts | 20% | EPA 5021 | b) |
| 1,3,5-Trimetylbensen | < 0.0050 | mg/kg Ts | 30% | EPA 5021 | b) |
| 1,3-Diklorbensen | < 0.0050 | mg/kg Ts | 15% | EPA 5021 | b) |
| 1,3-Diklorpropan | < 0.0050 | mg/kg Ts | 25% | EPA 5021 | b) |
| 1,3-Diklorpropen | < 0.0050 | mg/kg Ts | 25% | EPA 5021 | b) |
| 1,4-Diklorbensen | < 0.0050 | mg/kg Ts | 15% | EPA 5021 | b) |
| 2,2-Diklorpropan | < 0.0050 | mg/kg Ts | 30% | EPA 5021 | b) |
| 2-Klortoluen | < 0.0050 | mg/kg Ts | 30% | EPA 5021 | b) |
| 4-Klortoluen | < 0.0050 | mg/kg Ts | 30% | EPA 5021 | b) |
| Bensen | 0.91 | mg/kg Ts | 25% | EPA 5021 | b) |
| Brombensen | < 0.0050 | mg/kg Ts | 20% | EPA 5021 | b) |
| Bromdiklormetan | < 0.0050 | mg/kg Ts | 25% | EPA 5021 | b) |
| Bromklormetan | < 0.0050 | mg/kg Ts | 30% | EPA 5021 | b) |
| cis-1,2-Dikloreten | < 0.0050 | mg/kg Ts | 30% | EPA 5021 | b) |
| Dibromklormetan | < 0.0050 | mg/kg Ts | 25% | EPA 5021 | b) |
| Dibrommetan | < 0.0050 | mg/kg Ts | 30% | EPA 5021 | b) |
| Diklormetan | < 0.0050 | mg/kg Ts | 30% | EPA 5021 | b) |
| Etylbensen | 0.011 | mg/kg Ts | 20% | EPA 5021 | b) |
| Hexaklorbutadien (HCBD) | < 0.0050 | mg/kg Ts | 30% | EPA 5021 | b) |
| iso-Propylbensen | < 0.0050 | mg/kg Ts | 30% | EPA 5021 | b) |
| Klorbensen | < 0.0050 | mg/kg Ts | 25% | EPA 5021 | b) |
| m/p-Xylen | 0.013 | mg/kg Ts | 30% | EPA 5021 | b) |
| n-Butylbensen | < 0.0050 | mg/kg Ts | 30% | EPA 5021 | b) |
| o-Xylen | 0.0051 | mg/kg Ts | 30% | EPA 5021 | b) |
| p-Isopropyltoluen | 0.014 | mg/kg Ts | 30% | EPA 5021 | b) |
| Propylbensen | < 0.0050 | mg/kg Ts | 25% | EPA 5021 | b) |
| sec-Butylbensen | < 0.0050 | mg/kg Ts | 30% | EPA 5021 | b) |
| tert-Butylbensen | < 0.0050 | mg/kg Ts | 30% | EPA 5021 | b) |
| Tetrakloreten | < 0.0050 | mg/kg Ts | 20% | EPA 5021 | b) |
| Tetraklormetan | < 0.0050 | mg/kg Ts | 25% | EPA 5021 | b) |
| Toluen | 0.35 | mg/kg Ts | 20% | EPA 5021 | b) |
| trans-1,2-Dikloreten | < 0.0050 | mg/kg Ts | 30% | EPA 5021 | b) |
| trans-1,3-Diklorpropen | < 0.0050 | mg/kg Ts | 25% | EPA 5021 | b) |
| Tribrommetan | < 0.0050 | mg/kg Ts | 30% | EPA 5021 | b) |
| Triklorflourmetan (CFC-11) | < 0.0050 | mg/kg Ts | 30% | EPA 5021 | b) |
| Triklormetan | < 0.0050 | mg/kg Ts | 25% | EPA 5021 | b) |
| Aluminum Al | 940 | mg/kg Ts | 15% | SS028311 / ICP-AES | b) |
| Arsenik As | < 9.4 | mg/kg Ts | 25% | EN ISO 11885:2009 / SS 028311 utg 1 | b)* |
| Barium Ba | 16 | mg/kg Ts | 25% | EN ISO 11885:2009 / SS 028311 utg 1 | b)* |
| Bly Pb | 6.5 | mg/kg Ts | 25% | EN ISO 11885:2009 / SS 028311 utg 1 | b)* |
| Kadmium Cd | < 0.52 | mg/kg Ts | 25% | EN ISO 11885:2009 / SS 028311 utg 1 | b)* |
| Kobolt Co | < 2.4 | mg/kg Ts | 25% | EN ISO 11885:2009 / SS 028311 utg 1 | b)* |
| Koppar Cu | 22 | mg/kg Ts | 25% | EN ISO 11885:2009 / SS 028311 utg 1 | b)* |
| Krom Cr | 5 | mg/kg Ts | 25% | EN ISO 11885:2009 / SS 028311 utg 1 | b)* |
| Kvicksilver Hg | 0.049 | mg/kg Ts | 20% | SS028311mod/SS-EN ISO17852mod | b)* |
| Nickel Ni | 3 | mg/kg Ts | 25% | EN ISO 11885:2009 / SS 028311 utg 1 | b)* |
| Silver Ag | < 0.94 | mg/kg Ts | 25% | EN ISO 17294-2:2016 / SS 028311, utg 1 | b)* |
| Tenn Sn | 1.1 | mg/kg Ts | 25% | EN ISO 17294-2:2016 / SS 028311, utg 1 | b)* |
| Vanadin V | < 9.4 | mg/kg Ts | 25% | EN ISO 11885:2009 / SS 028311 utg 1 | b)* |
| Zink Zn | 67 | mg/kg Ts | 25% | EN ISO 11885:2009 / SS 028311 utg 1 | b)* |
| Summa Diklorfenoler | < 1.0 | mg/kg Ts | 20% | Internal Method LidMiljö.0A.01.17 | b)* |
| Summa Triklorfenol | < 1.0 | mg/kg Ts | 20% | Internal Method LidMiljö.0A.01.17 | b)* |
| Summa Tetraklorfenol | < 1.0 | mg/kg Ts | 20% | Internal Method LidMiljö.0A.01.17 | b)* |
| Pentaklorfenol | < 1.0 | mg/kg Ts | 20% | Internal Method LidMiljö.0A.01.17 | b)* |
| DDT-o,p | < 0.10 | mg/kg Ts | 20% | Internal Method LidMiljö.0A.01.17 | b)* |
| DDT,p,p'- | < 0.10 | mg/kg Ts | 20% | Internal Method LidMiljö.0A.01.17 | b)* |
| DDE,o,p- | < 0.10 | mg/kg Ts | 20% | Internal Method LidMiljö.0A.01.17 | b)* |
| DDE-p,p | < 0.10 | mg/kg Ts | 20% | Internal Method LidMiljö.0A.01.17 | b)* |
| HCH-alfa | < 0.10 | mg/kg Ts | 20% | Internal Method LidMiljö.0A.01.17 | b)* |
| HCH-beta | < 0.10 | mg/kg Ts | 20% | Internal Method LidMiljö.0A.01.17 | b)* |
| HCH-delta | < 0.10 | mg/kg Ts | 20% | Internal Method LidMiljö.0A.01.17 | b)* |
| HCH,gamma- (Lindane) | < 0.10 | mg/kg Ts | 20% | Internal Method LidMiljö.0A.01.17 | b)* |
| Hexaklorbensen | < 0.10 | mg/kg Ts | 20% | Internal Method LidMiljö.0A.01.17 | b)* |
| Endosulfan-alpha | < 0.10 | mg/kg Ts | 20% | Internal Method LidMiljö.0A.01.17 | b)* |
| Endosulfan-beta | < 0.10 | mg/kg Ts | 20% | Internal Method LidMiljö.0A.01.17 | b)* |
| Endosulfan-sulfate | < 0.10 | mg/kg Ts | 20% | Internal Method LidMiljö.0A.01.17 | b)* |
| Dieldrin | < 0.10 | mg/kg Ts | 20% | Internal Method LidMiljö.0A.01.17 | b)* |
| Endrin | < 0.10 | mg/kg Ts | 20% | Internal Method LidMiljö.0A.01.17 | b)* |
| PCB 28 | < 0.10 | mg/kg Ts | 20% | Internal Method LidMiljö.0A.01.17 | b)* |
| PCB 52 | < 0.10 | mg/kg Ts | 20% | Internal Method LidMiljö.0A.01.17 | b)* |
| PCB 101 | < 0.10 | mg/kg Ts | 20% | Internal Method LidMiljö.0A.01.17 | b)* |
| PCB 118 | < 0.10 | mg/kg Ts | 20% | Internal Method LidMiljö.0A.01.17 | b)* |
| PCB 153 | < 0.10 | mg/kg Ts | 20% | Internal Method LidMiljö.0A.01.17 | b)* |
| PCB 138 | < 0.10 | mg/kg Ts | 20% | Internal Method LidMiljö.0A.01.17 | b)* |
| PCB 180 | < 0.10 | mg/kg Ts | 20% | Internal Method LidMiljö.0A.01.17 | b)* |
| S:a PCB (7st) | < 0.40 | mg/kg Ts |  | Internal Method LidMiljö.0A.01.17 | b)* |
| N-nitroso-di-n-propylamin | < 0.10 | mg/kg Ts | 20% | Internal Method LidMiljö.0A.01.17 | b)* |
| Nitrobensen | < 0.10 | mg/kg Ts | 20% | Internal Method LidMiljö.0A.01.17 | b)* |
| Azobensen | < 0.10 | mg/kg Ts | 20% | Internal Method LidMiljö.0A.01.17 | b)* |
| N-nitrosodifenylamin | < 0.10 | mg/kg Ts | 20% | Internal Method LidMiljö.0A.01.17 | b)* |
| 2,6-Dinitrotoluen | < 0.10 | mg/kg Ts | 20% | Internal Method LidMiljö.0A.01.17 | b)* |
| 2,4-Dinitrotoluen | < 0.10 | mg/kg Ts | 20% | Internal Method LidMiljö.0A.01.17 | b)* |
| Bis(2-kloretyl)eter | < 0.10 | mg/kg Ts | 20% | Internal Method LidMiljö.0A.01.17 | b)* |
| Bis(2-kloroisopropyl)eter | < 0.10 | mg/kg Ts | 20% | Internal Method LidMiljö.0A.01.17 | b)* |
| Hexakloretan | < 0.10 | mg/kg Ts | 20% | Internal Method LidMiljö.0A.01.17 | b)* |
| Isophorone | < 0.10 | mg/kg Ts | 20% | Internal Method LidMiljö.0A.01.17 | b)* |
| Bis(2-kloretoxy)metan | < 0.10 | mg/kg Ts | 20% | Internal Method LidMiljö.0A.01.17 | b)* |
| 2-Klornaftalen | < 0.10 | mg/kg Ts | 20% | Internal Method LidMiljö.0A.01.17 | b)* |
| 4-Klorfenyl fenyleter | < 0.10 | mg/kg Ts | 20% | Internal Method LidMiljö.0A.01.17 | b)* |
| 4-Bromofenyl fenyleter | < 0.10 | mg/kg Ts | 20% | Internal Method LidMiljö.0A.01.17 | b)* |
| Pentaklorbensen | < 0.10 | mg/kg Ts | 20% | Internal Method LidMiljö.0A.01.17 | b)* |
| Dimetylftalat (DMP) | < 0.10 | mg/kg Ts | 20% | Internal Method LidMiljö.0A.01.17 | b)* |
| Dietylftalat | < 0.10 | mg/kg Ts | 20% | Internal Method LidMiljö.0A.01.17 | b)* |
| Di-n-butylftalat | < 0.10 | mg/kg Ts | 20% | Internal Method LidMiljö.0A.01.17 | b)* |
| Bensylbutylftalat | < 0.10 | mg/kg Ts | 20% | Internal Method LidMiljö.0A.01.17 | b)* |
| Di-(2-etylhexyl)ftalat | < 1.0 | mg/kg Ts | 20% | Internal Method LidMiljö.0A.01.17 | b)* |
| Di-n-oktylftalat | < 0.10 | mg/kg Ts | 20% | Internal Method LidMiljö.0A.01.17 | b)* |
| Metylkvicksilver | 0.83 | µg/kg Ts |  | EPA Method 1630 | c)* |
| 1-(3,4-Dichlorophenyl)-3-methylurea | <1.0 | µg/kg Ts | 27% | J. of Chromatogr. A, 1217 (2010) 2933–2939 mod. | a) |
| 1-(3,4-Dichlorophenyl)urea | <1.0 | µg/kg Ts | 27% | J. of Chromatogr. A, 1217 (2010) 2933–2939 mod. | a) |
| 2,4,5-T | <10 | µg/kg Ts | 27% | J. of Chromatogr. A, 1217 (2010) 2933–2939 mod. | a) |
| 2,4-D | <10 | µg/kg Ts | 27% | J. of Chromatogr. A, 1217 (2010) 2933–2939 mod. | a) |
| 2,4-Dichlorprop | <10 | µg/kg Ts | 27% | J. of Chromatogr. A, 1217 (2010) 2933–2939 mod. | a) |
| 2,6-Dichlorobenzamide | <10 | µg/kg Ts | 27% | J. of Chromatogr. A, 1217 (2010) 2933–2939 mod. | a) |
| Atrazine | <10 | µg/kg Ts | 27% | J. of Chromatogr. A, 1217 (2010) 2933–2939 mod. | a) |
| Atrazine-desethyl | <10 | µg/kg Ts | 27% | J. of Chromatogr. A, 1217 (2010) 2933–2939 mod. | a) |
| Atrazine-desisopropyl | <10 | µg/kg Ts | 27% | J. of Chromatogr. A, 1217 (2010) 2933–2939 mod. | a) |
| Bentazone | <10 | µg/kg Ts | 27% | J. of Chromatogr. A, 1217 (2010) 2933–2939 mod. | a) |
| Cyanazine | <10 | µg/kg Ts | 27% | J. of Chromatogr. A, 1217 (2010) 2933–2939 mod. | a) |
| Diuron | <1.0 | µg/kg Ts | 27% | J. of Chromatogr. A, 1217 (2010) 2933–2939 mod. | a) |
| Imazapyr | <10 | µg/kg Ts | 27% | J. of Chromatogr. A, 1217 (2010) 2933–2939 mod. | a) |
| Linuron | <10 | µg/kg Ts | 27% | J. of Chromatogr. A, 1217 (2010) 2933–2939 mod. | a) |
| MCPA | <10 | µg/kg Ts | 27% | J. of Chromatogr. A, 1217 (2010) 2933–2939 mod. | a) |
| Mecoprop | <10 | µg/kg Ts | 27% | J. of Chromatogr. A, 1217 (2010) 2933–2939 mod. | a) |
| Simazine | <10 | µg/kg Ts | 27% | J. of Chromatogr. A, 1217 (2010) 2933–2939 mod. | a) |
| Terbuthylazine | <10 | µg/kg Ts | 27% | J. of Chromatogr. A, 1217 (2010) 2933–2939 mod. | a) |

Non-accredited analyzes are marked with *

The measurement uncertainty, unless otherwise stated, is reported as extended measurement uncertainty with a coverage factor of 2. Increased reporting limit for PAHs, aliphatics and aromatics due to difficult sample matrix.

1. Eurofins Food & Feed Testing Sweden (Lidköping), SWEDEN, ISO/IEC 17025:2017 SWEDAC 1977
2. Eurofins Environment Testing Sweden AB, SWEDEN, ISO/IEC 17025:2017 SWEDAC 1125
3. IVL Svenska Miljöinstitutet AB, SWEDEN

**Table SM-2.** Dewatered autoclaved fiberbanks analysis report from accredited external laboratory. This fiberbanks used as a growing material.

| Analyte | Result | Unit | Measurement uncertainty | Metod/ref |  |
| --- | --- | --- | --- | --- | --- |
| Torrsubstans | 96.1 | % | 5% | SS-EN 12880:2000 | b) |
| Alifater >C8-C10 | < 20 | mg/kg Ts | 35% | SPI 2011 | b)* |
| Alifater >C10-C12 | < 20 | mg/kg Ts | 30% | SPI 2011 | b)* |
| Alifater >C12-C16 | < 20 | mg/kg Ts | 30% | SPI 2011 | b)* |
| Alifater >C16-C35 | 3000 | mg/kg Ts | 30% | SPI 2011 | b)* |
| Aromater >C8-C10 | < 40 | mg/kg Ts | 30% | SPI 2011 | b)* |
| Aromater >C10-C16 | < 4.0 | mg/kg Ts | 20% | SPI 2011 | b)* |
| Aromater >C16-C35 | 2 | mg/kg Ts | 25% | SPI 2011 | b)* |
| Metylpyren/fluorantener | < 2.0 | mg/kg Ts | 25% | SPI 2011 | b)* |
| Metylkrysener/benzo(a)antracener | < 2.0 | mg/kg Ts | 25% | SPI 2011 | b)* |
| Bens(a)antracen | < 0.14 | mg/kg Ts | 25% | ISO 18287:2008 mod | b) |
| Krysen | < 0.14 | mg/kg Ts | 25% | ISO 18287:2008 mod | b) |
| Benso(b,k)fluoranten | < 0.14 | mg/kg Ts | 25% | ISO 18287:2008 mod | b) |
| Benso(a)pyren | < 0.14 | mg/kg Ts | 25% | ISO 18287:2008 mod | b) |
| Indeno(1,2,3-cd)pyren | < 0.14 | mg/kg Ts | 25% | ISO 18287:2008 mod | b) |
| Dibens(a,h)antracen | < 0.14 | mg/kg Ts | 30% | ISO 18287:2008 mod | b) |
| Naftalen | 2.3 | mg/kg Ts | 25% | ISO 18287:2008 mod | b) |
| Acenaftylen | 0.17 | mg/kg Ts | 40% | ISO 18287:2008 mod | b) |
| Acenaften | 0.39 | mg/kg Ts | 25% | ISO 18287:2008 mod | b) |
| Fluoren | 0.14 | mg/kg Ts | 30% | ISO 18287:2008 mod | b) |
| Fenantren | 0.37 | mg/kg Ts | 25% | ISO 18287:2008 mod | b) |
| Antracen | < 0.14 | mg/kg Ts | 25% | ISO 18287:2008 mod | b) |
| Fluoranten | 0.2 | mg/kg Ts | 25% | ISO 18287:2008 mod | b) |
| Pyren | 0.31 | mg/kg Ts | 25% | ISO 18287:2008 mod | b) |
| Benso(g,h,i)perylen | < 0.14 | mg/kg Ts | 25% | ISO 18287:2008 mod | b) |
| Summa PAH med låg molekylvikt | 2.9 | mg/kg Ts |  |  | b) |
| Summa PAH med medelhög molekylvikt | 1.1 | mg/kg Ts |  |  | b) |
| Summa PAH med hög molekylvikt | < 0.49 | mg/kg Ts |  |  | b) |
| Summa cancerogena PAH | < 0.42 | mg/kg Ts |  |  | b) |
| Summa övriga PAH | 4 | mg/kg Ts |  |  | b) |
| Summa totala PAH16 | 4.4 | mg/kg Ts |  |  | b) |
| 1,1,1,2-Tetrakloretan | < 0.0050 | mg/kg Ts | 20% | EPA 5021 | b) |
| 1,1,1-Trikloretan | < 0.0050 | mg/kg Ts | 25% | EPA 5021 | b) |
| 1,1,2-Trikloretan | < 0.0050 | mg/kg Ts | 30% | EPA 5021 | b) |
| 1,1,2-Trikloreten | < 0.0050 | mg/kg Ts | 20% | EPA 5021 | b) |
| 1,1-Dikloretan | < 0.0050 | mg/kg Ts | 30% | EPA 5021 | b) |
| 1,1-Dikloreten | < 0.0050 | mg/kg Ts | 30% | EPA 5021 | b) |
| 1,1-Diklorpropen | < 0.0050 | mg/kg Ts | 25% | EPA 5021 | b) |
| 1,2,3-Triklorbensen | < 0.0050 | mg/kg Ts | 30% | EPA 5021 | b) |
| 1,2,3-Triklorpropan | < 0.0050 | mg/kg Ts | 25% | EPA 5021 | b) |
| 1,2,4-Triklorbensen | < 0.0050 | mg/kg Ts | 20% | EPA 5021 | b) |
| 1,2,4-Trimetylbensen | < 0.0050 | mg/kg Ts | 30% | EPA 5021 | b) |
| 1,2-Dibrometan | < 0.0050 | mg/kg Ts | 25% | EPA 5021 | b) |
| 1,2-Diklorbensen | < 0.0050 | mg/kg Ts | 15% | EPA 5021 | b) |
| 1,2-Dikloretan | < 0.0050 | mg/kg Ts | 25% | EPA 5021 | b) |
| 1,2-Diklorpropan | < 0.0050 | mg/kg Ts | 20% | EPA 5021 | b) |
| 1,3,5-Trimetylbensen | < 0.0050 | mg/kg Ts | 30% | EPA 5021 | b) |
| 1,3-Diklorbensen | < 0.0050 | mg/kg Ts | 15% | EPA 5021 | b) |
| 1,3-Diklorpropan | < 0.0050 | mg/kg Ts | 25% | EPA 5021 | b) |
| 1,3-Diklorpropen | < 0.0050 | mg/kg Ts | 25% | EPA 5021 | b) |
| 1,4-Diklorbensen | < 0.0050 | mg/kg Ts | 15% | EPA 5021 | b) |
| 2,2-Diklorpropan | < 0.0050 | mg/kg Ts | 30% | EPA 5021 | b) |
| 2-Klortoluen | < 0.0050 | mg/kg Ts | 30% | EPA 5021 | b) |
| 4-Klortoluen | < 0.0050 | mg/kg Ts | 30% | EPA 5021 | b) |
| Bensen | 0.17 | mg/kg Ts | 25% | EPA 5021 | b) |
| Brombensen | < 0.0050 | mg/kg Ts | 20% | EPA 5021 | b) |
| Bromdiklormetan | < 0.0050 | mg/kg Ts | 25% | EPA 5021 | b) |
| Bromklormetan | < 0.0050 | mg/kg Ts | 30% | EPA 5021 | b) |
| cis-1,2-Dikloreten | < 0.0050 | mg/kg Ts | 30% | EPA 5021 | b) |
| Dibromklormetan | < 0.0050 | mg/kg Ts | 25% | EPA 5021 | b) |
| Dibrommetan | < 0.0050 | mg/kg Ts | 30% | EPA 5021 | b) |
| Diklormetan | 0.016 | mg/kg Ts | 30% | EPA 5021 | b) |
| Etylbensen | 0.0076 | mg/kg Ts | 20% | EPA 5021 | b) |
| Hexaklorbutadien (HCBD) | < 0.0050 | mg/kg Ts | 30% | EPA 5021 | b) |
| iso-Propylbensen | < 0.0050 | mg/kg Ts | 30% | EPA 5021 | b) |
| Klorbensen | < 0.0050 | mg/kg Ts | 25% | EPA 5021 | b) |
| m/p-Xylen | 0.0092 | mg/kg Ts | 30% | EPA 5021 | b) |
| n- Butylbensen | < 0.0050 | mg/kg Ts | 30% | EPA 5021 | b) |
| o- Xylen | < 0.0050 | mg/kg Ts | 30% | EPA 5021 | b) |
| p- Isopropyltoluen | 0.014 | mg/kg Ts | 30% | EPA 5021 | b) |
| Propylbensen | < 0.0050 | mg/kg Ts | 25% | EPA 5021 | b) |
| sec-Butylbensen | < 0.0050 | mg/kg Ts | 30% | EPA 5021 | b) |
| tert-Butylbensen | < 0.0050 | mg/kg Ts | 30% | EPA 5021 | b) |
| Tetrakloreten | < 0.0050 | mg/kg Ts | 20% | EPA 5021 | b) |
| Tetraklormetan | < 0.0050 | mg/kg Ts | 25% | EPA 5021 | b) |
| Toluen | 0.45 | mg/kg Ts | 20% | EPA 5021 | b) |
| trans-1,2-Dikloreten | < 0.0050 | mg/kg Ts | 30% | EPA 5021 | b) |
| trans-1,3-Diklorpropen | < 0.0050 | mg/kg Ts | 25% | EPA 5021 | b) |
| Tribrommetan | < 0.0050 | mg/kg Ts | 30% | EPA 5021 | b) |
| Triklorflourmetan (CFC-11) | < 0.0050 | mg/kg Ts | 30% | EPA 5021 | b) |
| Triklormetan | 0.49 | mg/kg Ts | 25% | EPA 5021 | b) |
| Aluminum Al | 870 | mg/kg Ts | 15% | SS028311 / ICP-AES | b) |
| Arsenik As | < 9.4 | mg/kg Ts | 25% | EN ISO 11885:2009 / SS 028311 utg 1 | b)* |
| Barium Ba | 16 | mg/kg Ts | 25% | EN ISO 11885:2009 / SS 028311 utg 1 | b)* |
| Bly Pb | 5.5 | mg/kg Ts | 25% | EN ISO 11885:2009 / SS 028311 utg 1 | b)* |
| Kadmium Cd | < 0.53 | mg/kg Ts | 25% | EN ISO 11885:2009 / SS 028311 utg 1 | b)* |
| Kobolt Co | < 2.4 | mg/kg Ts | 25% | EN ISO 11885:2009 / SS 028311 utg 1 | b)* |
| Koppar Cu | 24 | mg/kg Ts | 25% | EN ISO 11885:2009 / SS 028311 utg 1 | b)* |
| Krom Cr | 5.2 | mg/kg Ts | 25% | EN ISO 11885:2009 / SS 028311 utg 1 | b)* |
| Kvicksilver Hg | < 0.047 | mg/kg Ts | 20% | SS028311mod/SS-EN ISO17852mod | b)* |
| Nickel Ni | 3 | mg/kg Ts | 25% | EN ISO 11885:2009 / SS 028311 utg 1 | b)* |
| Silver Ag | < 0.94 | mg/kg Ts | 25% | EN ISO 17294-2:2016 / SS 028311, utg 1 | b)* |
| Tenn Sn | 0.97 | mg/kg Ts | 25% | EN ISO 17294-2:2016 / SS 028311, utg 1 | b)* |
| Vanadin V | < 9.4 | mg/kg Ts | 25% | EN ISO 11885:2009 / SS 028311 utg 1 | b)* |
| Zink Zn | 68 | mg/kg Ts | 25% | EN ISO 11885:2009 / SS 028311 utg 1 | b)* |
| Summa Diklorfenoler | < 1.0 | mg/kg Ts | 20% | Internal Method LidMiljö.0A.01.17 | b)* |
| Summa Triklorfenol | < 1.0 | mg/kg Ts | 20% | Internal Method LidMiljö.0A.01.17 | b)* |
| Summa Tetraklorfenol | < 1.0 | mg/kg Ts | 20% | Internal Method LidMiljö.0A.01.17 | b)* |
| Pentaklorfenol | < 1.0 | mg/kg Ts | 20% | Internal Method LidMiljö.0A.01.17 | b)* |
| DDT-o,p | < 0.10 | mg/kg Ts | 20% | Internal Method LidMiljö.0A.01.17 | b)* |
| DDT,p,p'- | < 0.10 | mg/kg Ts | 20% | Internal Method LidMiljö.0A.01.17 | b)* |
| DDE,o,p- | < 0.10 | mg/kg Ts | 20% | Internal Method LidMiljö.0A.01.17 | b)* |
| DDE-p,p | < 0.10 | mg/kg Ts | 20% | Internal Method LidMiljö.0A.01.17 | b)* |
| HCH-alfa | < 0.10 | mg/kg Ts | 20% | Internal Method LidMiljö.0A.01.17 | b)* |
| HCH-beta | < 0.10 | mg/kg Ts | 20% | Internal Method LidMiljö.0A.01.17 | b)* |
| HCH-delta | < 0.10 | mg/kg Ts | 20% | Internal Method LidMiljö.0A.01.17 | b)* |
| HCH,gamma- (Lindane) | < 0.10 | mg/kg Ts | 20% | Internal Method LidMiljö.0A.01.17 | b)* |
| Hexaklorbensen | < 0.10 | mg/kg Ts | 20% | Internal Method LidMiljö.0A.01.17 | b)* |
| Endosulfan-alpha | < 0.10 | mg/kg Ts | 20% | Internal Method LidMiljö.0A.01.17 | b)* |
| Endosulfan-beta | < 0.10 | mg/kg Ts | 20% | Internal Method LidMiljö.0A.01.17 | b)* |
| Endosulfan-sulfate | < 0.10 | mg/kg Ts | 20% | Internal Method LidMiljö.0A.01.17 | b)* |
| Dieldrin | < 0.10 | mg/kg Ts | 20% | Internal Method LidMiljö.0A.01.17 | b)* |
| Endrin | < 0.10 | mg/kg Ts | 20% | Internal Method LidMiljö.0A.01.17 | b)* |
| PCB 28 | < 0.10 | mg/kg Ts | 20% | Internal Method LidMiljö.0A.01.17 | b)* |
| PCB 52 | < 0.10 | mg/kg Ts | 20% | Internal Method LidMiljö.0A.01.17 | b)* |
| PCB 101 | < 0.10 | mg/kg Ts | 20% | Internal Method LidMiljö.0A.01.17 | b)* |
| PCB 118 | < 0.10 | mg/kg Ts | 20% | Internal Method LidMiljö.0A.01.17 | b)* |
| PCB 153 | < 0.10 | mg/kg Ts | 20% | Internal Method LidMiljö.0A.01.17 | b)* |
| PCB 138 | < 0.10 | mg/kg Ts | 20% | Internal Method LidMiljö.0A.01.17 | b)* |
| PCB 180 | < 0.10 | mg/kg Ts | 20% | Internal Method LidMiljö.0A.01.17 | b)* |
| S:a PCB (7st) | < 0.40 | mg/kg Ts |  | Internal Method LidMiljö.0A.01.17 | b)* |
| N-nitroso-di-n-propylamin | < 0.10 | mg/kg Ts | 20% | Internal Method LidMiljö.0A.01.17 | b)* |
| Nitrobensen | < 0.10 | mg/kg Ts | 20% | Internal Method LidMiljö.0A.01.17 | b)* |
| Azobensen | < 0.10 | mg/kg Ts | 20% | Internal Method LidMiljö.0A.01.17 | b)* |
| N-nitrosodifenylamin | < 0.10 | mg/kg Ts | 20% | Internal Method LidMiljö.0A.01.17 | b)* |
| 2,6-Dinitrotoluen | < 0.10 | mg/kg Ts | 20% | Internal Method LidMiljö.0A.01.17 | b)* |
| 2,4-Dinitrotoluen | < 0.10 | mg/kg Ts | 20% | Internal Method LidMiljö.0A.01.17 | b)* |
| Bis(2-kloretyl)eter | < 0.10 | mg/kg Ts | 20% | Internal Method LidMiljö.0A.01.17 | b)* |
| Bis(2-kloroisopropyl)eter | < 0.10 | mg/kg Ts | 20% | Internal Method LidMiljö.0A.01.17 | b)* |
| Hexakloretan | < 0.10 | mg/kg Ts | 20% | Internal Method LidMiljö.0A.01.17 | b)* |
| Isophorone | < 0.10 | mg/kg Ts | 20% | Internal Method LidMiljö.0A.01.17 | b)* |
| Bis(2-kloretoxy)metan | < 0.10 | mg/kg Ts | 20% | Internal Method LidMiljö.0A.01.17 | b)* |
| 2-Klornaftalen | < 0.10 | mg/kg Ts | 20% | Internal Method LidMiljö.0A.01.17 | b)* |
| 4-Klorfenyl fenyleter | < 0.10 | mg/kg Ts | 20% | Internal Method LidMiljö.0A.01.17 | b)* |
| 4-Bromofenyl fenyleter | < 0.10 | mg/kg Ts | 20% | Internal Method LidMiljö.0A.01.17 | b)* |
| Pentaklorbensen | < 0.10 | mg/kg Ts | 20% | Internal Method LidMiljö.0A.01.17 | b)* |
| Dimetylftalat (DMP) | < 0.10 | mg/kg Ts | 20% | Internal Method LidMiljö.0A.01.17 | b)* |
| Dietylftalat | < 0.10 | mg/kg Ts | 20% | Internal Method LidMiljö.0A.01.17 | b)* |
| Di-n-butylftalat | < 0.10 | mg/kg Ts | 20% | Internal Method LidMiljö.0A.01.17 | b)* |
| Bensylbutylftalat | < 0.10 | mg/kg Ts | 20% | Internal Method LidMiljö.0A.01.17 | b)* |
| Di-(2-etylhexyl)ftalat | < 1.0 | mg/kg Ts | 20% | Internal Method LidMiljö.0A.01.17 | b)* |
| Di-n-oktylftalat | < 0.10 | mg/kg Ts | 20% | Internal Method LidMiljö.0A.01.17 | b)* |
| Metylkvicksilver | 3.1 | µg/kg Ts |  | EPA Method 1630 | c)* |
| 1-(3,4-Dichlorophenyl)-3-methylurea | <1.0 | µg/kg Ts | 27% | J. of Chromatogr. A, 1217 (2010) 2933–2939 mod. | a) |
| 1-(3,4-Dichlorophenyl)urea | <1.0 | µg/kg Ts | 27% | J. of Chromatogr. A, 1217 (2010) 2933–2939 mod. | a) |
| 2,4,5-T | <10 | µg/kg Ts | 27% | J. of Chromatogr. A, 1217 (2010) 2933–2939 mod. | a) |
| 2,4-D | <10 | µg/kg Ts | 27% | J. of Chromatogr. A, 1217 (2010) 2933–2939 mod. | a) |
| 2,4-Dichlorprop | <10 | µg/kg Ts | 27% | J. of Chromatogr. A, 1217 (2010) 2933–2939 mod. | a) |
| 2,6-Dichlorobenzamide | <10 | µg/kg Ts | 27% | J. of Chromatogr. A, 1217 (2010) 2933–2939 mod. | a) |
| Atrazine | <10 | µg/kg Ts | 27% | J. of Chromatogr. A, 1217 (2010) 2933–2939 mod. | a) |
| Atrazine-desethyl | <10 | µg/kg Ts | 27% | J. of Chromatogr. A, 1217 (2010) 2933–2939 mod. | a) |
| Atrazine-desisopropyl | <10 | µg/kg Ts | 27% | J. of Chromatogr. A, 1217 (2010) 2933–2939 mod. | a) |
| Bentazone | <10 | µg/kg Ts | 27% | J. of Chromatogr. A, 1217 (2010) 2933–2939 mod. | a) |
| Cyanazine | <10 | µg/kg Ts | 27% | J. of Chromatogr. A, 1217 (2010) 2933–2939 mod. | a) |
| Diuron | <1.0 | µg/kg Ts | 27% | J. of Chromatogr. A, 1217 (2010) 2933–2939 mod. | a) |
| Imazapyr | <10 | µg/kg Ts | 27% | J. of Chromatogr. A, 1217 (2010) 2933–2939 mod. | a) |
| Linuron | <10 | µg/kg Ts | 27% | J. of Chromatogr. A, 1217 (2010) 2933–2939 mod. | a) |
| MCPA | <10 | µg/kg Ts | 27% | J. of Chromatogr. A, 1217 (2010) 2933–2939 mod. | a) |
| Mecoprop | <10 | µg/kg Ts | 27% | J. of Chromatogr. A, 1217 (2010) 2933–2939 mod. | a) |
| Simazine | <10 | µg/kg Ts | 27% | J. of Chromatogr. A, 1217 (2010) 2933–2939 mod. | a) |
| Terbuthylazine | <10 | µg/kg Ts | 27% | J. of Chromatogr. A, 1217 (2010) 2933–2939 mod. | a) |

Non-accredited analyzes are marked with *

The measurement uncertainty, unless otherwise stated, is reported as extended measurement uncertainty with a coverage factor of 2. Increased reporting limit for PAHs, aliphatics and aromatics due to difficult sample matrix.

1. Eurofins Food & Feed Testing Sweden (Lidköping), SWEDEN, ISO/IEC 17025:2017 SWEDAC 1977
2. Eurofins Environment Testing Sweden AB, SWEDEN, ISO/IEC 17025:2017 SWEDAC 1125
3. IVL Svenska Miljöinstitutet AB, SWEDEN

| Species | Vanadium | Chromium | Cobalt | Nickel | Copper | Zinc | Arsenic | Cadmium | Lead |
| --- | --- | --- | --- | --- | --- | --- | --- | --- | --- |
| *B. adusta* | 0.3 ± 0.1 | 1.43 ± 0.23 | 0.19 ± 0.07 | 1.2 ± 0.2 | 6.52 ± 0.48 | 42.62 ± 2.62 | <0.0001 | 0.35 ± 0.05 | 1.71 ± 0.21 |
| *C. muraii* | 0.5 ± 0.1 | 2.38 ± 0.08 | 0.32 ± 0.05 | 2 ± 0.3 | 10.86 ± 0.86 | 70.98 ± 3.98 | 0.21 ± 0.03 | 0.58 ± 0.08 | 2.85 ± 0.35 |
| *D. crustulinus* | 1.05 ±0.05 | 5.02 ± 0.02 | 0.68 ± 0.10 | 4.22 ± 0.22 | 22.89 ± 0.89 | 149.58 ± 4.58 | 0.44 ± 0.04 | 1.21 ± 0.21 | 6.01 ± 0.5 |
| *G. applanatum* | 0.63 ± 0.12 | 3.04 ± 0.24 | 0.41 ± 0.11 | 2.55 ± 0.25 | 13.86 ± 0.8 | 90.54 ± 5.54 | 0.27 ± 0.07 | 0.73 ± 0.11 | 3.64 ± 0.14 |
| *H. annosum* | 1.02 ± 0.14 | 4.87 ± 0.17 | 0.65 ± 0.15 | 4.09 ± 0.22 | 22.2 ± 1.2 | 145.01 ± 2.01 | 0.42 ± 0.02 | 1.18 ± 0.18 | 5.83 ± 0.45 |
| *H. tabacina* | 1.05 ± 0.05 | 5.05 ± 0.12 | 0.68 ± 0.13 | 4.24 ± 0.24 | 23.01 ± 0.21 | 150.32 ± 4.32 | 0.44 ± 0.04 | 1.22 ± 0.22 | 6.04 ± 0.2 |
| *L. sulphureus* | 1.11 ±0.11 | 5.34 ± 0.13 | 0.72 ± 0.12 | 4.48 ± 0.4 | 24.34 ± 0.31 | 159 ± 9.45 | 0.47 ± 0.07 | 1.29 ± 0.29 | 6.39 ± 0.39 |
| *P. ferrugineofuscus* | 0.93 ± 0.03 | 4.47 ± 0.15 | 0.6 ± 0.2 | 3.76 ± 026 | 20.38 ± 0.93 | 133.15 ± 3.15 | 0.39 ± 0.09 | 1.08 ± 0.03 | 5.35 ± 0.35 |
| *P. punctatus* | 1.03 ± 0.03 | 4.95 ± 0.15 | 0.66 ± 0.11 | 4.15 ± 0.15 | 22.55 ± 0.55 | 147.3 ± 5.3 | 0.43 ± 0.03 | 1.19 ± 0.19 | 5.92 ± 0.3 |
| *P. tremellosa* | 1.3 ± 0.4 | 6.23 ± 0.23 | 0.84 ± 0.1 | 5.23 ± 0.23 | 28.39 ± 0.94 | 185.52 ± 2.53 | 0.54 ± 0.04 | 1.5 ± 0.3 | 7.45 ± 0.45 |
| *P. gigantea* | 0.57 ± 0.07 | 2.75 ± 0.15 | 0.37 ± 0.07 | 2.31 ± 0.31 | 12.55 ± 0.55 | 81.97 ± 3.97 | 0.24 ± 0.04 | 0.66 ± 0.1 | 3.29 ± 0.29 |
| *S. odora* | 0.64 ± 0.05 | 3.05 ± 0.25 | 0.41 ± 0.1 | 2.56 ± 0.4 | 13.91 ± 0.91 | 90.87 ± 4.87 | 0.27 ± 0.07 | 0.74 ± 0.04 | 3.65 ± 0.4 |
| *S. sanguinolentum* | 1.02 ± 0.02 | 4.87 ± 0.87 | 0.65 ± 0.07 | 4.09 ± 0.09 | 22.17 ± 0.17 | 144.86 ± 5. 86 | 0.42 ± 0.04 | 1.17 ± 0.17 | 5.82 ± 0.2 |
| *T. hirsuta* | 0.86 ± 0.06 | 4.12 ± 0.32 | 0.55 ± 0.05 | 3.46 ± 0.46 | 18.76 ± 0.26 | 122.58 ± 3.01 | 0.36 ± 0.05 | 0.99 ± 0.2 | 4.93 ± 0.4 |
| *T. ochracea* | 0.27 ± 0.03 | 1.31 ± 0.31 | 0.18 ± 0.05 | 1.1 ± 0.1 | 5.96 ± 0.5 | 38.96 ± 2.96 | 0.11 ± 0.01 | 0.32 ± 0.04 | 1.57 ± 0.3 |
| Fiberbanks | 3.35 | 62.49 | 0.465 | 4.09 | 18.41 | 51.44 | 0.65 | 0.27 | 4.51 |

**Table SM-3.** Metal concentration (n=3) in fungal tissue (mg/kg), and in fiberbank.

| **Species** | **V** | **Cr** | **Co** | **Ni** | **Cu** | **Zn** | **As** | **Cd** | **Pb** |
| --- | --- | --- | --- | --- | --- | --- | --- | --- | --- |
| *B. adusta* | 0.09 | 0.02 | 0.41 | 0.29 | 0.35 | 0.83 | 0 | 1.3 | 0.38 |
| *C. muraii* | 0.15 | 0.04 | 0.69 | 0.49 | 0.59 | 1.38 | 0.32 | 2.15 | 0.63 |
| *D. crustulinus* | 0.31 | 0.08 | 1.46 | 1.03 | 1.24 | 2.91 | 0.68 | 4.48 | 1.33 |
| *G. applanatum* | 0.19 | 0.05 | 0.88 | 0.62 | 0.75 | 1.76 | 0.42 | 2.7 | 0.81 |
| *H. annosum* | 0.3 | 0.08 | 1.4 | 1 | 1.21 | 2.82 | 0.65 | 4.37 | 1.29 |
| *H. tabacina* | 0.31 | 0.08 | 1.46 | 1.04 | 1.25 | 2.92 | 0.68 | 4.52 | 1.34 |
| *L. sulphureus* | 0.33 | 0.09 | 1.55 | 1.1 | 1.32 | 3.09 | 0.72 | 4.78 | 1.42 |
| *P. ferrugineofuscus* | 0.28 | 0.07 | 1.29 | 0.92 | 1.11 | 2.59 | 0.6 | 4 | 1.19 |
| *P. punctatus* | 0.31 | 0.08 | 1.42 | 1.01 | 1.22 | 2.86 | 0.66 | 4.41 | 1.31 |
| *P. tremellosa* | 0.39 | 0.1 | 1.81 | 1.28 | 1.54 | 3.61 | 0.83 | 5.56 | 1.65 |
| *P. gigantea* | 0.17 | 0.04 | 0.8 | 0.56 | 0.68 | 1.59 | 0.37 | 2.44 | 0.73 |
| *S. odora* | 0.19 | 0.05 | 0.88 | 0.63 | 0.76 | 1.77 | 0.42 | 2.74 | 0.81 |
| *S. sanguinolentum* | 0.3 | 0.08 | 1.4 | 1 | 1.2 | 2.82 | 0.65 | 4.33 | 1.29 |
| *T. hirsuta* | 0.26 | 0.07 | 1.18 | 0.85 | 1.02 | 2.38 | 0.55 | 3.67 | 1.09 |
| *T. ochracea* | 0.08 | 0.02 | 0.39 | 0.27 | 0.32 | 0.76 | 0.17 | 1.19 | 0.35 |

**Table SM-4.** Bioconcentration factor of WRF for the targeted elements (n=3).

**Table SM-5.** Results from ANOVA.

| Elements | F-value | Pr(>F) | Significance level | Between Groups DF | Within Groups DF |
| --- | --- | --- | --- | --- | --- |
| Arsenic | 27.57 | 2.65e-13 | <0.001 | 14 | 30 |
| Chromium | 76.49 | <2e-16 | <0.001 | 14 | 30 |
| Zinc | 2424.00 | <2e-16 | <0.001 | 14 | 30 |
| Vanadium | 17.65 | 9.41e-11 | <0.001 | 14 | 30 |
| Copper | 278.2 | <2e-16 | <0.001 | 14 | 30 |
| Cobalt | 10.58 | 4.82e-08 | <0.001 | 14 | 30 |
| Lead | 81.00 | <2e-16 | <0.001 | 14 | 30 |
| Nickel | 62.51 | <2e-16 | <0.001 | 14 | 30 |
| Cadmium | 13.22 | 3.44e-09 | <0.001 | 14 | 30 |
